# A new paradigm for generating high-quality cardiac pacemaker cells from mouse pluripotent stem cells

**DOI:** 10.1038/s41392-024-01942-w

**Published:** 2024-09-06

**Authors:** Zheyi Lin, Bowen Lin, Chengwen Hang, Renhong Lu, Hui Xiong, Junyang Liu, Siyu Wang, Zheng Gong, Mingshuai Zhang, Desheng Li, Guojian Fang, Jie Ding, Xuling Su, Huixin Guo, Dan Shi, Duanyang Xie, Yi Liu, Dandan Liang, Jian Yang, Yi-Han Chen

**Affiliations:** 1 State Key Laboratory of Cardiovascular Diseases, Shanghai East Hospital, School of Medicine, Tongji University, Shanghai, 200120 China; 2 Shanghai Arrhythmia Research Center, Shanghai East Hospital, School of Medicine, Tongji University, Shanghai, 200120 China; 3 Department of Cardiology, Shanghai East Hospital, School of Medicine, Tongji University, Shanghai, 200120 China; 4 Shanghai Frontiers Center of Nanocatalytic Medicine, Shanghai, 200092 China; 5 Department of Pathology and Pathophysiology, School of Medicine, Tongji University, Shanghai, 200092 China; 6 Clinical Center for Heart Disease Research, Tongji University, Shanghai, 200092 China; 7 Department of Cell Biology, School of Medicine, Tongji University, Shanghai, 200092 China; 8 Department of Cardiology, The Second Hospital of Shanxi Medical University, Taiyuan, 030001 China; 9 Research Units of Origin and Regulation of Heart Rhythm, Chinese Academy of Medical Sciences, Shanghai, 200092 China

**Keywords:** Differentiation, Cardiology, Stem-cell differentiation

## Abstract

Cardiac biological pacing (BP) is one of the future directions for bradyarrhythmias intervention. Currently, cardiac pacemaker cells (PCs) used for cardiac BP are mainly derived from pluripotent stem cells (PSCs). However, the production of high-quality cardiac PCs from PSCs remains a challenge. Here, we developed a cardiac PC differentiation strategy by adopting dual PC markers and simulating the developmental route of PCs. First, two PC markers, *Shox2* and *Hcn4*, were selected to establish *Shox2:EGFP; Hcn4:mCherry* mouse PSC reporter line. Then, by stepwise guiding naïve PSCs to cardiac PCs following naïve to formative pluripotency transition and manipulating signaling pathways during cardiac PCs differentiation, we designed the FSK method that increased the yield of SHOX2^+^; HCN4^+^ cells with typical PC characteristics, which was 12 and 42 folds higher than that of the embryoid body (EB) and the monolayer M10 methods respectively. In addition, the in vitro cardiac PCs differentiation trajectory was mapped by single-cell RNA sequencing (scRNA-seq), which resembled in vivo PCs development, and ZFP503 was verified as a key regulator of cardiac PCs differentiation. These PSC-derived cardiac PCs have the potential to drive advances in cardiac BP technology, help with the understanding of PCs (patho)physiology, and benefit drug discovery for PC-related diseases as well.

## Introduction

Under physiological conditions, the cardiac conduction system (CCS) sends electrical impulses spontaneously and periodically to the atria and ventricles to trigger the heart to produce regular contractile activity.^1,2^ The CCS consists of the sinoatrial node (SAN), the atrioventricular node (AVN), the right and left bundle branches, and the Purkinje fiber network. The electrical impulses of the CCS originate from parenchymal cells known as PCs in SAN.^2,3^ A variety of factors such as myocardial ischemia, myocarditis, cardiomyopathy, hypertension, heart failure, gene mutation and cardiac degeneration can cause cardiac PC-related disorders, which manifest bradyarrhythmia, tachyarrhythmia, cardiogenic syncope, cardiogenic shock and even sudden cardiac death.^4^ At present, the treatment of severe cardiac PC-related disorders mainly relies on implantable electronic cardiac pacemakers (ECP), but ECP does not correct defects in the cardiac PCs themselves.^5^ Moreover, although great advances have been made since its first introduction to clinical practice in the 1950s, inevitably, ECP-related complications are still not uncommon, such as pneumothorax, cardiac perforation, and infection.^5^ Cardiac BP is one of the alternative strategies to arrhythmia treatment.^6^ Currently, cardiac BP can be achieved by gene-based, cell-based, hybrid gene-cell, and cellular reprogramming-based approaches.^7^ Cardiac BP has been the focus and hot spot of cardiac pacemaking in recent years.

However, the development of cardiac BP technology was restricted by a number of factors, the most central of which was the availability of high-quality cardiac PCs. The number of cardiac PCs in SAN is extremely low, with only about 10,000 PCs in human SAN. In view of this, stem cell differentiation and somatic reprogramming are two alternative approaches to obtaining cardiac PCs, although both methods produce heterogeneous cell populations. To purify cardiac PCs from other cell types, either an antibody against a specific PC marker or a fluorescence reporter cell line indicating specific PC gene expression is needed.

By lineage tracing during embryo development, a set of genes, including *Hcn4*, *Tbx3, Tbx5*, *Tbx18*, *Shox2*, and *Isl1*, have been validated to relatively specifically express in the forming SAN either transiently or constitutively.^1,3^ Among these cardiac PC-specific expression genes, the short stature homeobox 2 (SHOX2) and the hyperpolarization and cyclic nucleotide 4 (HCN4) come to our attention. SHOX2 plays a pivotal role in SAN development and function.^8,9^ Mechanistically, SHOX2 prevents the formation of working myocardium by inhibiting *Nkx2-5* expression while activating *Hcn4*, *Isl1*, and *Tbx3* expression.^1,3^ Meanwhile, HCN4 is a widely accepted PC marker, which is responsible for generating funny current (*I*_*f*_), the main driver of automatic electrical activity in cardiac PCs.^10^ So far, four members of HCN have been identified, *Hcn1–4*, which express differentially in the heart, with *Hcn4* highly expressed in the SAN followed by *Hcn1*.^11^ In *Hcn4* knock-out (KO) mice, a 70% reduction in *I*_*f*_ current was recorded with an overall 60% reduction of spontaneous heart rate.^12^ The properties of *Shox2* and *Hcn4* may be utilized to target cardiac PCs during differentiation.

PSCs, either derived from the epiblast of early embryos (embryonic stem cells, ESCs) or reprogrammed from somatic cells (induced pluripotent stem cells, iPSCs), can provide a cell source for cardiac BP by in vitro differentiation.^13^ Both cardiac PCs and working cardiomyocytes have been derived from PSCs. Despite a series of key advances in cardiac PCs differentiation, the production of cardiac PCs is less sophisticated than that of working cardiomyocytes in terms of the quality of resulting cells.^14–17^

Epiblast in blastocyst is the founder of the body, which generates three germ layers and germ cells. In parallel, naïve ESCs have been derived from epiblast in blastocyst (embryonic days 3.5–4.5, E3.5–E4.5) while primed PSCs, termed epiblast stem cells (EpiSCs) can be derived from post-implantation embryo epiblast (E5.5–E6.5).^18^ Unlike naïve ESCs, EpiSCs display restricted pluripotency and more heterogenous differentiation potential.^19,20^ It has been reported that in vitro, the differentiation efficiency from naïve ESCs directly is not as high as expected.^21^ Therefore, it has been postulated that there are intermediate stages between naïve and primed pluripotent states, and as naïve epiblast exits pluripotency, it undergoes a capacitation stage to acquire full differentiation potential, which has been corroborated by both in vivo analysis and in vitro derivation of formative stem cells (FSCs) from E5.5–E6.0 post-implantation epiblast.^22^ Apart from the potential to generate primordial germ cells (PGCs),^23^ FSCs can differentiate into cell types representing three germ layers with higher efficiency.^22,24^

Here, by applying PC-specific markers and simulating the developmental route of PCs, we developed an integrated strategy to generate cardiac PCs from PSCs. *Shox2:EGFP; Hcn4:mCherry* mESC reporter line was constructed to track the in vitro differentiation of cardiac PCs. By following the naïve to formative transition and manipulating the signaling pathways involved in cardiac PCs development, we established a simple and efficient cardiac PCs differentiation method to produce more than 20% SHOX2^+^; HCN4^+^ cardiac PCs. Both in vivo and in vitro characterization revealed the similarity between PSC-derived cardiac PCs and bona fide PCs. With scRNA-seq, we mapped the differentiation trajectory from naïve ESCs to cardiac PCs and validated *Zfp503*, a target of RA,^25^ as a key factor regulating cardiac PCs differentiation.

## Results

### Construction of *Shox2:EGFP; Hcn4:mCherry* mESC reporter line

**Fig. 1:**
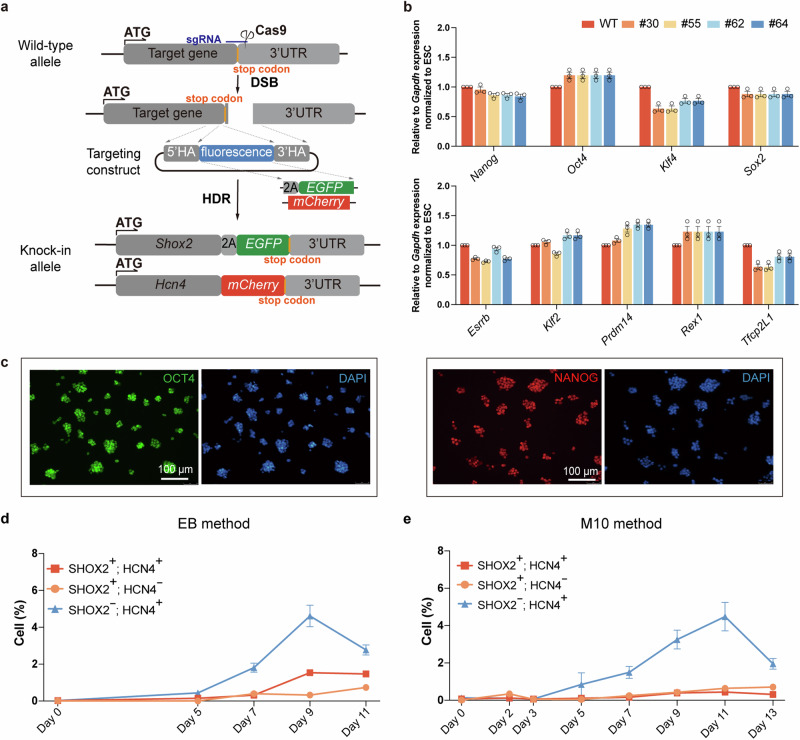
**Construction of** ***Shox2:EGFP; Hcn4:mCherry*** **mESC reporter line and cardiac PCs differentiation**. **a** Schematic overview of the construction of *Shox2:EGFP; Hcn4:mCherry* mESC reporter line. *2A-EGFP* and *mCherry* were inserted into 3’UTR of *Shox2* and *Hcn4*, respectively, by CRISPR/Cas9 system. HA, homologous arm. **b** qRT-PCR analysis of pluripotency marker gene expression of *Shox2:EGFP; Hcn4:mCherry* mESCs cultured in 2i/LIF, relative to *Gapdh* expression, normalized to WT E14TG2a mESCs (2i/LIF). HM *Shox2:EGFP; Hcn4:mCherry* mESC clones cultured in 2i/LIF at least three passages. Data were presented as means ± standard error of the mean (SEM) from biological triplicates (*n* = 3). **c** Immunofluorescence of *Shox2:EGFP; Hcn4:mCherry* mESCs cultured in 2i/LIF. Pluripotency markers: OCT4 (green) and NANOG (red). Scale bar: 100 µm. **d** Flow cytometry analysis of fluorescent subpopulations during cardiac PCs differentiation from days 0 to 11 by the EB method. Red square, SHOX2^+^; HCN4^+^ cells; yellow circle, SHOX2^+^; HCN4^−^ cells; blue triangle, SHOX2^−^; HCN4^+^ cells. Data were presented as means ± SEM from biological triplicates (*n* = 3). **e** Flow cytometry analysis of fluorescent subpopulations during cardiac PCs differentiation from days 0 to 13 by the M10 method. Red square, SHOX2^+^; HCN4^+^ cells; yellow circle, SHOX2^+^; HCN4^−^ cells; blue triangle, SHOX2^−^; HCN4^+^ cells. Data were presented as means ± SEM from biological triplicates (*n* = 3)

*Shox2* and *Hcn4* have been identified as two key genes contributing to cardiac PCs’ gene regulatory network and electrophysiological properties, respectively.^26,27^ Overexpressing HCN4 in human iPSCs and mESCs-derived cardiomyocytes enables the cells to possess partial cardiac PC characteristics and exogenous SHOX2 expression promoted the differentiation of ESCs into cardiac PCs.^16,28,29^ To more precisely monitor the rising of cardiac PCs from PSCs and obtain functional PCs for cardiac BP, we set to construct an mESC reporter line with *EGFP* and *mCherry* fluorescence genes knocked into *Shox2* and *Hcn4* 3’ untranslated region (3’UTR) respectively by CRISPR/Cas9 mediated homology-directed repair (HDR) (Fig. 1a). In order to minimize side impact, we selected the protospacer-adjacent motif (PAM) on the 3’UTR of target genes close to the last exon to design single guide RNA (sgRNA) (Supplementary Fig. S1a). First, *mCherry* was introduced into 3’UTR of the *Hcn4* locus in wild-type (WT) E14TG2a mESCs. From the 50 clones we picked, by genomic PCR, 30 clones were identified as homozygous (HM) *Hcn4:mCherry* mESCs. We then selected and expanded one HM *Hcn4:mCherry* clone for *Shox2:EGFP* targeting. We picked 64 clones for genotyping and identified five HM *Shox2:EGFP; Hcn4:mCherry* mESC clones (Supplementary Fig. S1b, c). Further Sanger sequencing of PCR products verified the in-frame sequence of the target gene and fluorescence reporter (Supplementary Fig. S1d). Furthermore, we analyzed some putative off-targets by PCR and sequencing, which revealed no mutations in these sites (Supplementary Fig. S1e, f).

While culturing the clones in naïve 2i/LIF medium, the colonies manifested dome-shaped morphologies, and no visible fluorescence was observed under a fluorescence microscope (Supplementary Fig. S1g). At the transcription level, most of the naïve pluripotency markers, including *Oct4*, *Sox2, Nanog*, and others, maintain their expression similar to those in WT mESCs (Fig. 1b). Immunostaining also confirmed homogenous expression of NANOG and OCT4 in *Shox2:EGFP; Hcn4:mCherry* mESCs (Fig. 1c). These results indicated that we have successfully established the *Shox2:EGFP; Hcn4:mCherry* mESC reporter line.

### Validation of the credibility of the mESC reporter line for cardiac PCs differentiation

Traditionally, the EB method has been widely used to examine the differentiation potential of PSCs in vitro.^17,30,31^ We set out to determine the credibility of the mESC reporter line during cardiac PCs differentiation using the EB method by monitoring fluorescent cells (Supplementary Fig. S2a). First, a drop of M10 medium containing 1000 mESCs was hung on the lid of a tissue culture dish. Five days later, the EBs formed and were plated into the gelatin-coated plate with WNT inhibitor IWR1-endo (days 5–7). On day 7, very few SHOX2^+^; HCN4^+^ cells emerged (Supplementary Fig. S2b). On day 9, about 1.6% of dual positive cells were determined by flow cytometry analysis, which lasted with a similar scale (1.31%–1.84%) in the whole differentiation process of the EB method (Fig. 1d and Supplementary Fig. S2c, d). Notably, on day 13, the expression pattern of *Shox2:EGFP; Hcn4:mCherry* EBs was similar to that of WT EBs. High expression of cardiac lineage marker *Tnnt2*, atrial markers *Myh6* and *Myl7*, and ventricular marker *Myl2* revealed that atrial-like cardiomyocytes (ALCMs) and ventricular-like cardiomyocytes (VLCMs) were the main cardiac cell types in EBs, whereas cardiac PC markers (*Shox2* and *Hcn4*)^32,33^ and cardiac PC-associated developmental gene *Tbx18* were lowly expressed (Supplementary Fig. S2e–g). These data suggested by observing the fluorescent cells during differentiation, the cardiac PCs may be identified for further analysis.

The small quantity of cardiac PCs derived from the EB method encouraged us to develop a new, simple, and efficient method for cardiac PCs generation. A monolayer human PSC differentiation protocol with sequential WNT activation and inactivation has been applied to produce working cardiomyocytes with more than 90% efficiency.^34^ However, the efficiency of the monolayer cardiac PCs differentiation method from mouse PSCs is still very poor and needs to be optimized. We modified a previous EB method^30^ by culturing *Shox2:EGFP; Hcn4:mCherry* mESCs in serum/LIF, which resembles a primed pluripotent state, as a monolayer (called M10 method) (Supplementary Fig. S3a). Cardiac PCs differentiation was induced by M10 medium supplemented with Vitamin C (Vc) for 3 days (days 0–3) and the cells were treated with WNT activator CHIR99021 (CHIR) for 24 h (days 2–3), then switched to serum-free medium for 2 days (days 3–5) and supplemented WNT inhibitor IWP2 for 2 days (days 5–7). On day 7, some HCN4^+^ cells emerged, but no SHOX2^+^ cells were observed (Fig. 1e and Supplementary Fig. S3b, c). After further differentiation, three subpopulations of cells with SHOX2^+^; HCN4^−^, SHOX2^−^; HCN4^+^, and SHOX2^+^; HCN4^+^ were identified in culture, all with extremely low ratios though (Fig. 1e and Supplementary Fig. S3b). The flow cytometry analysis detected only 0.52% and 0.41% SHOX2^+^; HCN4^+^ cells at days 11 and 13, respectively, during the differentiation period (Fig. 1e and Supplementary Fig. S3b, d), even less than the EB method (Supplementary Fig. S2c). Taken together, consistent with previous reports,^30^ both the EB and M10 methods produced a low number of cardiac PCs, indicating the importance of developing a new cardiac PCs differentiation strategy.

### The transition from naïve to formative pluripotency rendered efficient cardiac PCs differentiation of mESC reporter line

**Fig. 2:**
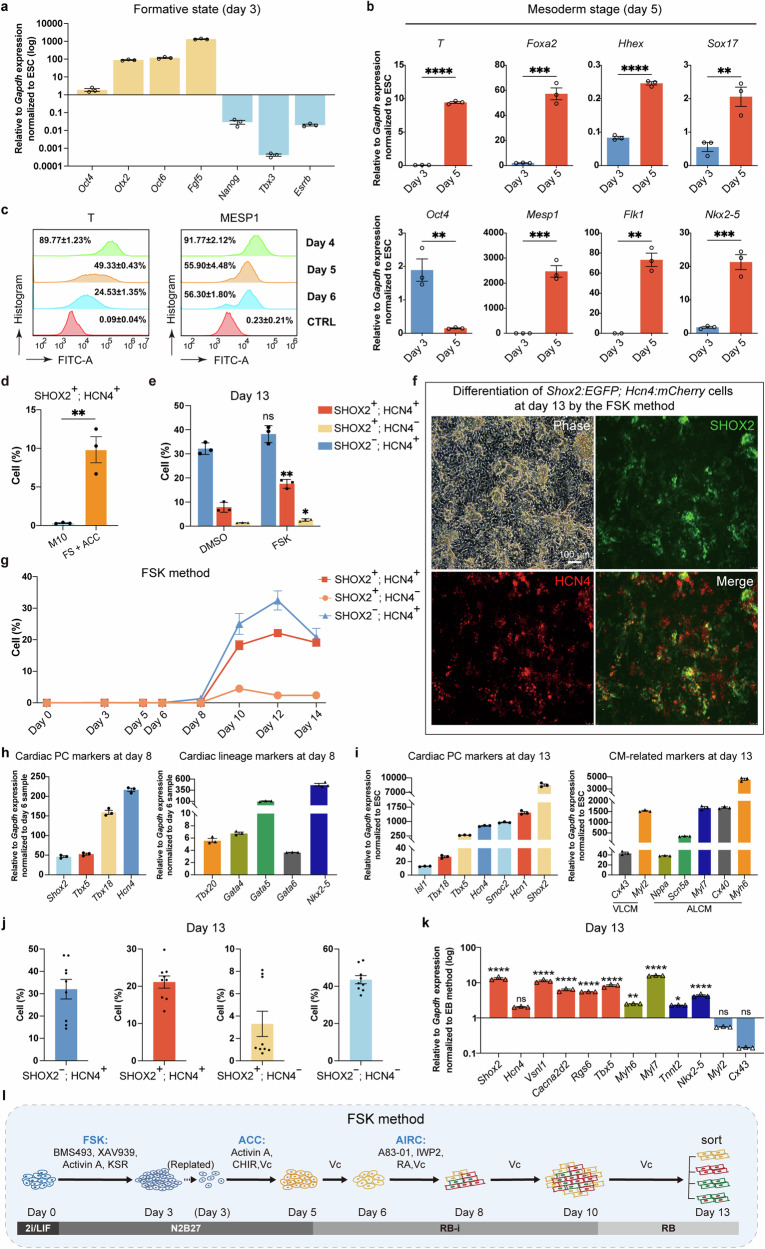
**Development of the FSK method for efficient cardiac PCs generation**. **a** qRT-PCR analysis revealed that the FSK method induced the transition from naïve to formative state on differentiation day 3. Relative to *Gapdh* expression, normalized to *Shox2:EGFP; Hcn4:mCherry* mESCs (2i/LIF). Pluripotency marker: *Oct4*; formative markers: *Otx2, Oct6*, and *Fgf5*; naïve markers: *Nanog*, *Tbx3*, and *Esrrb*. Data were presented as means ± SEM from technical triplicates (*n* = 3). **b** qRT-PCR analysis revealed that ACC induced the commitment of FSCs into mesoderm on differentiation day 5. Relative to *Gapdh* expression, normalized to *Shox2:EGFP; Hcn4:mCherry* mESCs (2i/LIF). Primitive streak markers: *T* and *Foxa2*; endoderm markers: *Hhex* and *Sox17;* pluripotency marker: *Oct4*; mesoderm markers: *Mesp1* and *Flk1*; cardiac lineage marker: *Nkx2-5*. Data were presented as means ± SEM from technical triplicates (*n* = 3). **c** Flow cytometry analysis of T^+^ and MESP1^+^ cells during differentiation from days 4 to 6. CTRL, control represented differentiated cells collected on day 5 stained with the fluorescent secondary antibody only. Data were presented as means ± SEM from biological triplicates (*n* = 3). **d** Flow cytometry analysis of SHOX2^+^; HCN4^+^ cells on differentiation day 13 after induction by the M10 method or FS + ACC induction. ‘FS + ACC’ represented the FS transition (days 0–3) and mesoderm induction (ACC induction, days 5–7). Data were presented as means ± SEM from biological triplicates (*n* = 3). **e** Comparison of cardiac PCs differentiation efficiency on day 13. In addition to FS + ACC, AIRC induction was combined to treat cells from days 6–8 in the FSK group, while only an equal volume of dimethyl sulfoxide (DMSO) was added in the DMSO group. Red, SHOX2^+^; HCN4^+^ cells; yellow, SHOX2^+^; HCN4^−^ cells; blue, SHOX2^−^; HCN4^+^ cells. Data were presented as means ± SEM from biological triplicates (*n* = 3). **f** Representative live cell images displayed the expression pattern of HCN4 and SHOX2 by the FSK method on day 13 using *Shox2:EGFP; Hcn4:mCherry* mESC reporter line. Scale bar: 100 µm. **g** Percentage of three fluorescent subpopulations in the process of the FSK method. Red square, SHOX2^+^; HCN4^+^ cells; yellow circle, SHOX2^+^; HCN4^−^ cells; blue triangle, SHOX2^−^; HCN4^+^ cells. Data were presented as means ± SEM from biological triplicates (*n* = 3). **h** Expression pattern of cardiac-related markers in day 8 differentiated cells by the FSK method. Experiments were performed on *Shox2:EGFP; Hcn4:mCherry* mESC reporter line. Relative to *Gapdh* expression, normalized to day 6 differentiated cells. Cardiac PC markers: *Shox2, Tbx5, Tbx18*, and *Hcn4;* early cardiac lineage markers: *Tbx20, Gata4, Gata5, Gata6*, and *Nkx2-5*. Data were presented as means ± SEM from technical triplicates (*n* = 3). **i** Transcriptional level of cardiac-related markers in day 13 differentiated cells by the FSK method. Relative to *Gapdh* expression, normalized to *Shox2:EGFP; Hcn4:mCherry* mESCs (2i/LIF). Cardiac PC markers: *Isl1*, *Tbx18, Tbx5, Hcn4, Smoc2, Hcn1*, and *Shox2;* VLCM markers: *Cx43* and *Myl2*; ALCM markers: *Nppa, Scn5a, Myl7, Cx40*, and *Myh6*. Data were presented as means ± SEM from technical triplicates (*n* = 3). **j** Flow cytometry analysis of fluorescent subpopulations on differentiation day 13. Data were presented as means ± SEM from 9 biological samples of 3 independent experiments. **k** Differences in the expression of cardiac-related markers between the FSK and EB methods. qRT-PCR analysis of gene expression on day 13 differentiated cells. Relative to *Gapdh* expression, normalized to day 13 *Shox2:EGFP; Hcn4:mCherry* EBs. Cardiac PC markers: *Shox2, Hcn4, Vsnl1, Cacna2d2, Rgs6*, and *Tbx5* (orange)*;* ALCM markers: *Myh6* and *Myl7* (green); cardiac lineage markers: *Tnnt2* and *Nkx2-5* (deep blue); VLCM markers: *Myl2* and *Cx43* (light blue). Data were presented as means ± SEM from technical triplicates (*n* = 3). **l** Diagram of the FSK method. mESCs cultured in 2i/LIF were used as seed cells. RB-i, RPMI1640 plus B27 minus insulin; RB, RPMI1640 plus B27. *P* values were calculated using a two-tailed student *t*-test to compare two groups of data (**b**, **d**, **e**). Comparisons between multiple groups in the FSK and EB methods were performed with the one-way ANOVA test (**k**). Statistical significance was indicated as follows: ns, not significant; *P* < 0.05 (*); *P* < 0.01 (**); *P* < 0.001 (***); *P* < 0.0001 (****)

As naïve PSCs are more homogenous than primed PSCs in terms of transcriptome and epigenome,^35^ and FSCs are prone to lineage commitment,^22^ we plated naïve 2i/LIF mESCs as seeds for cardiac PCs differentiation. The formative state (FS) transition was achieved by treating naïve mESCs with Activin A, tankyrase inhibitor XAV939, pan-retinoic acid receptor inverse agonist BMS493 and KnockOut serum replacement (KSR) for 3 days, evidenced by existence of pluripotency factor *Oct4*, increased expression of formative markers, *Otx2, Oct6, Fgf5*, and absence of naïve markers, *Nanog*, *Tbx3*, and *Esrrb* (Fig. 2a), which was similar to previous studies.^22,24^ The PSCs proliferated significantly during the 72-h FS transition. After this process, ubiquitous OTX2 expression in FSCs was observed (Supplementary Fig. S4a, b).

Vc is included in media for both the EB and M10 methods (Supplementary Figs. S2a and 3a). Apart from its anti-oxidant effects, Vc has been shown as an important epigenetic modifier that plays a critical role in cell fate transition.^36^ We, therefore, added Vc after mesoderm commitment from the FS. Subsequently, after 2-day (days 3–5) Activin A, CHIR and Vc (called ACC) treatment, loss of pluripotency marker *Oct4*, upregulation of primitive streak markers *Brachyury* (*T*) and *Foxa2*, lateral cardiac mesodermal lineage-specific genes (*Mesp1* and *Flk1*), and early cardiac lineage marker *Nkx2-5* were detected, whereas the expression of endoderm markers, *Sox17* and *Hhex*, was relatively low (Fig. 2b). We further examined the protein expression of T and MESP1 during mesoderm induction. The majority of cells on day 4 were T^+^ and MESP1^+^, which declined gradually with further induction, suggesting the efficient differentiation of cardiac mesodermal cells (Fig. 2c). Then, after continuous culture, 9.83 ± 5.81% SHOX2^+^; HCN4^+^ cells were generated on day 13 from ACC induction treated FS transition (FS + ACC), which was 32 folds more than the M10 method (Fig. 2d).

### Small molecules targeting WNT, Activin/Nodal/TGF-β, and RA pathways further increased cardiac PCs differentiation efficiency of mESC reporter line

As activation of RA and inhibition of WNT and Activin/Nodal/TGF-β signaling pathways have been reported to induce cardiac PCs differentiation,^14,37–39^ we first applied RA, A83-01 (TGF-β type I receptor inhibitor), and IWP2 individually during the differentiation of cardiac mesoderm into cardiac PCs from days 6 to 8. Five days after induction, the fluorescent cells were analyzed by flow cytometry, within the concentrations of tested small molecules, both RA and A83-01 increased SHOX2^−^; HCN4^+^ cells without effects on SHOX2^+^; HCN4^+^ cells. For IWP2, about an 11% increase of SHOX2^−^; HCN4^+^ cells were detected at high concentration (10 μM), however, there was about a 3% reduction of SHOX2^+^; HCN4^+^ cells as well (Supplementary Fig. S4c).

We then combined A83-01, IWP2, and RA together with Vc (called AIRC) for differentiation induction. Following FS conversion through FSK medium and combining with ACC induction and AIRC induction, we named the new cardiac PCs differentiation protocol as FSK method.

Although there was a slight increase in SHOX2^−^; HCN4^+^ cells (5%), an about 2.3-fold increase in SHOX2^+^; HCN4^+^ cells was achieved, reaching 17.57 ± 3.50%, compared with control (Fig. 2e), suggesting the synergetic effects of these signaling pathways in promoting cardiac PCs differentiation. A wide range of fluorescent cells was observed under the microscope on day 13 (Fig. 2f). We monitored the dynamic change of fluorescent cells by flow cytometry, which showed gradual increase of SHOX2^−^; HCN4^+^ and SHOX2^+^; HCN4^+^ cells from day 8 and reached apex on day 12, with 32.43 ± 3.03% and 22.1 ± 1.10% respectively, then SHOX2^−^; HCN4^+^ cells began to decrease, whereas SHOX2^+^; HCN4^+^ cells were relatively stable. On the other hand, less than 6% SHOX2^+^; HCN4^−^ cells were produced during the whole process (Fig. 2g and Supplementary Fig. S4d, e).

Similarly, the expression of a set of cardiac PCs markers significantly increased from day 8 after AIRC induction, although the markers for cardiac lineage were detected as well (Fig. 2h). After continuous culture, besides *Tbx5*, *Tbx18*, *Shox2*, and *Hcn4*, cardiac PC-related transcription factor (TF) *Isl1*, ion channel *Hcn1*, gap junction *Smoc2* and cardiomyocyte-related markers (*Cx43*, *Myl2*, *Nppa*, *Scn5a*, *Myl7*, *Cx40*, and *Myh6*) displayed high transcription level at day 13 (Fig. 2i). SHOX2^−^; HCN4^+^ and SHOX2^+^; HCN4^+^ cells represented more than half of the population (32.02 ± 4.36% and 21.14 ± 1.63%, respectively), while the production of SHOX2^+^; HCN4^−^ cells was much lower (3.31 ± 1.13%) at day 13 (Fig. 2j). Compared with the EB method, differentiated cells on day 13 by the FSK method displayed higher expression of cardiac PC (*Shox2, Hcn4, Vsnl1, Cacna2d2, Rgs6*, and *Tbx5*), ALCM (*Myh6* and M*yl7*) and cardiac lineage markers (*Tnnt2* and *Nkx2-5*), while lower expression of VLCM markers (*Myl2* and *Cx43*) was detected. These data indicated that higher efficiency of cardiac lineage differentiation and enrichment of cardiac PCs and ALCMs in differentiated cells were induced by this new FSK method (Fig. 2k).

We thus established a new paradigm for cardiac PCs differentiation, the FSK method, which is composed of naïve to formative pluripotency transition, sequential mesodermal, and cardiac PCs induction (Fig. 2l).

### Homogenous formative PSCs acted as the main determinant of cardiac PCs differentiation

To determine the effects of a homogenous FS on cardiac PCs differentiation, we applied the FSK method on the M10 method by ACC induction on serum/LIF ESCs or replacing IWP2 plus Vc with AIRC (Supplementary Fig. S4f). While AIRC did not show beneficial effects, ACC induction displayed a 3.4-fold increase in SHOX2^+^; HCN4^+^ cells (1.83 ± 0.84%) compared with the control (0.54 ± 0.21%) (Supplementary Fig. S4g). Overall, the modification of the M10 method did not raise the cardiac PCs productivity closer to the FSK method, implying that the employment of naïve mESCs played a decisive role.

We then performed cardiac PCs differentiation of WT E14TG2a mESCs by applying the FSK method. By immunofluorescence, compared to the M10 method, cells with high expression of cardiac PCs markers HCN4, SHOX2, and VSNL1 were widely distributed in day 13 differentiated cells accompanied by cTnT^+^ and NKX2-5^+^ cells, further validating the robustness of this method (Supplementary Fig. S4h). These results implicated that our FSK method might induce a higher ratio of FSCs to differentiate into cardiac PCs.

### SHOX2^+^; HCN4^+^ cells displayed cardiac PC characteristics

**Fig. 3:**
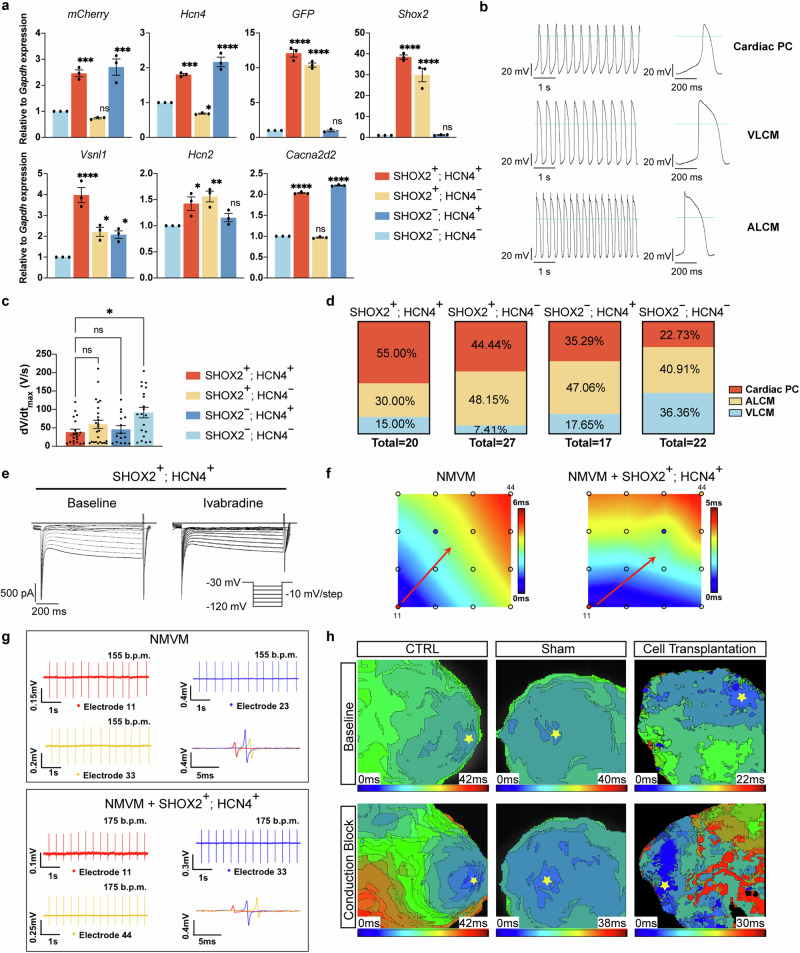
**SHOX2**^**+**^**; HCN4**^**+**^ **cells displayed cardiac PC characteristics.** **a** qRT-PCR analysis of the expression of cardiac lineage and cardiac PC marker genes in sorted cells. Relative to *Gapdh* expression, normalized to SHOX2^−^; HCN4^−^ cells. *mCherry* and *EGFP* were closely correlated to *Hcn4* and *Shox2* expression, respectively. Cardiac PC-related TF marker: *Shox2*; cardiac PC-related ion channels: *Hcn4*, *Hcn2*, and *Cacna2d2*; cardiac PC enriched gene: *Vsnl1*. Red, SHOX2^+^; HCN4^+^ cells; yellow, SHOX2^+^; HCN4^−^ cells; deep blue, SHOX2^−^; HCN4^+^ cells; light blue, SHOX2^−^; HCN4^−^ cells. Data were presented as means ± SEM from technical triplicates (*n* = 3). **b** Representative sAP recording of single cells differentiated from *Shox2:EGFP; Hcn4:mCherry* mESCs via the FSK method. **c** The maximum uplink speed of four different subpopulations. Red, SHOX2^+^; HCN4^+^ cells (*n* = 20); yellow, SHOX2^+^; HCN4^−^ cells (*n* = 27); deep blue, SHOX2^−^; HCN4^+^ cells (*n* = 17); light blue, SHOX2^−^; HCN4^−^ cells (*n* = 22). Data were presented as means ± SEM, *n* indicated the number of recorded cells. **d** The proportion of different cell types according to AP forms in the sorted subpopulations. Red, cardiac PC; yellow, ALCM; light blue, VLCM. **e** The *I*_*f*_ current recording of SHOX2^+^; HCN4^+^ cells. Baselines were recorded before adding 1 µM ivabradine into Tyrode’s solution with 1 mM Ba^2+^. **f** Activation maps of electrical signal propagation revealed that impulses generated from cardiac PC aggregates. Colormap indicated the origin of impulses generated from the electrode 11. Cardiac PC aggregate was laid next to electrode 11 and propagated electrical signal to pace NMVMs. Red arrows represented the directions of impulse propagation. **g** Representative raw traces showed the addition of cardiac PC aggregate increased the beating rate. Representative raw traces of the monolayer NMVMs with cardiac PC aggregate were recorded at electrodes 11, 33, and 44 with the speed of 175 b.p.m. **h** Differentiated cells from the FSK method acted as an ectopic pacemaker in rat atrioventricular conduction block model. Yellow stars indicated the pacemaking sites. Cells were sorted and collected on day 13 differentiation (**a**–**e**). sAP recording and *I*_*f*_ current recording were performed on HEKA EPC-10 amplifier (**b**–**e**). Comparisons between multiple groups were performed with a one-way ANOVA test (**a**) and Kruskal-Wallis rank sum test (**c**). Statistical significance was indicated as follows: ns not significant; *P* < 0.05 (*); *P* < 0.01 (**); *P* < 0.001 (***); *P* < 0.0001 (****)

The fluorescence reporter cell line is beneficial for specific subtype purification and further functional characterization. To identify the cell types representing cardiac PCs, we sorted differentiated cells on day 13. The sorted cell morphologies displayed spider-like or spindle-like features (Supplementary Fig. S5a, b and Supplementary Video 1). Gene expression profile revealed that besides *Shox2* and *Hcn4*, SHOX2^+^; HCN4^+^ cells enriched other cardiac PC markers, including *Vsnl1*, *Hcn2*, and *Cacna2d2* (Fig. 3a and Supplementary Fig. S5c). The high expression of these ion channel and calcium-binding protein-encoding genes is the basis for the cardiac PCs’ function.^33,40^ *Tbx5* and *Rgs6*, as cardiac PC markers, were also enriched in SHOX2^+^; HCN4^+^ cells, suggesting the cardiac PC regulatory network was activated (Supplementary Fig. S5c). For SHOX2^+^; HCN4^−^ cells, the upregulation of *Hcn2* may compensate for the relatively low *Hcn4* expression (Fig. 3a). Although *Cacna2d2*, *Tbx5*, and *Rgs6* were detected in SHOX2^−^; HCN4^+^ cells, lower expression of *Vsnl1*^32^ and lack of *Shox2*^41^ may compromise their cardiac PCs identity (Fig. 3a and Supplementary Fig. S5c). Meanwhile, in the context of the wide presence of early cardiac lineage markers (*Myh6* and *Nkx2-5*) and cardiac cytoskeleton gene *Tnnt2*, we speculated that day 13 differentiated cells were similar to embryonic cardiac cells (Supplementary Fig. S5c).

Cardiac PCs have distinct electrophysiological properties. After cell sorting, we first performed a spontaneous action potential (sAP) assay and recorded diverse sAP forms representing three cell types, including cardiac PC, ALCM, and VLCM (Fig. 3b). In general, SHOX2^+^; HCN4^+^ cells displayed a fast spontaneous emission rate, slow maximum uplink speed, more positive maximum diastolic potential (MDP) and small action potential amplitude (APA) compared with other subpopulations (Fig. 3c and Supplementary Fig. S5d). The maximum uplink rate of SHOX2^+^; HCN4^+^ cells (38.53 ± 7.92 V/s) was 42.08%, 64.88%, and 84.44% of SHOX2^−^; HCN4^−^ (91.55 ± 14.23 V/s), SHOX2^+^; HCN4^−^ (59.39 ± 11.44 V/s) and SHOX2^−^; HCN4^+^ (45.63 ± 10.28 V/s) cells respectively (Fig. 3c). According to the results of sAP recording, on average, 45.31% of the SHOX2, HCN4 single and double positive cells displayed the functional sAP of cardiac PCs (Fig. 3d), which meant a large portion of cardiac PCs can be obtained from the FSK method. 55.00% of SHOX2^+^; HCN4^+^ cells showed cardiac PC sAP, which was the highest among the four sorted populations. A similar ratio of ALCM sAP exists in two subpopulations (48.15% in SHOX2^+^; HCN4^−^ cells and 47.06% in SHOX2^−^; HCN4^+^ cells) while 36.36% of SHOX2^−^; HCN4^−^ cells were considered as VLCMs (Fig. 3d). To further characterize the electrophysiological properties of SHOX2^+^; HCN4^+^ cells, the *I*_*f*_ current was also recorded in SHOX2^+^; HCN4^+^ cells which were sensitive to ivabradine (Fig. 3e). We further detected the effect of cAMP on *I*_*f*_ current in SHOX2^+^; HCN4^+^ cells. Similar to SAN cells, *I*_*f*_ currents showed an increase in response to cAMP activity (Supplementary Fig. S5e, f). Meanwhile, SHOX2^+^; HCN4^+^ cells could maintain cardiac PC characteristics at least up to day 30 with similar maximum uplink rate and ivabradine-sensitive *I*_*f*_ currents (Supplementary Fig. S5g–i). In all, these data indicated that SHOX2^+^; HCN4^+^ cells were more similar to cardiac PCs.

Next, we examined the pacemaker potential of SHOX2^+^; HCN4^+^ cell aggregates in vitro. The cells were plated into a low-attachment U-bottom 96-well plate and centrifuged to make aggregates (Supplementary Fig. S5j). The aggregates were then seeded onto neonatal mouse ventricular cardiomyocytes (NMVMs), and the pacing activity of cardiac PC aggregates were assessed by multiple microelectrode array (MEA) assays. Generally, monolayer NMVMs alone have multiple initiation sites. In the presence of SHOX2^+^; HCN4^+^ cardiac PCs aggregates, a dominant pacemaker stably paced NMVMs, increasing the pace from 155 beats per minute (b.p.m.) to 175 b.p.m., and generated more rhythmic beats; higher FPD_max_ (FPD, field potential duration) and larger spike amplitude were detected as well (Fig. 3f, g). These results indicated that SHOX2^+^; HCN4^+^ cells functioned as cardiac BP in vitro.

Finally, we tested the pacemaker activity of differentiated cells in vivo (Supplementary Fig. S5k). We injected 1–2 × 10^6^ differentiated cells into the apex of the rat heart with daily immunosuppressive treatment. Optical mapping revealed that the implanted cells paced the rat’s left ventricle for a short period during drug-induced short-term atrioventricular conduction block (Fig. 3h). Subsequent paraffin section confirmed the presence of implanted cells in the heart by co-localization of SHOX2 and HCN4:mCherry, which may form a connection with host ventricular cardiomyocytes via CX43 (Supplementary Fig. S5l, m).

### Cardiac PC cluster was identified by scRNA-seq during in vitro cardiac PCs differentiation

**Fig. 4:**
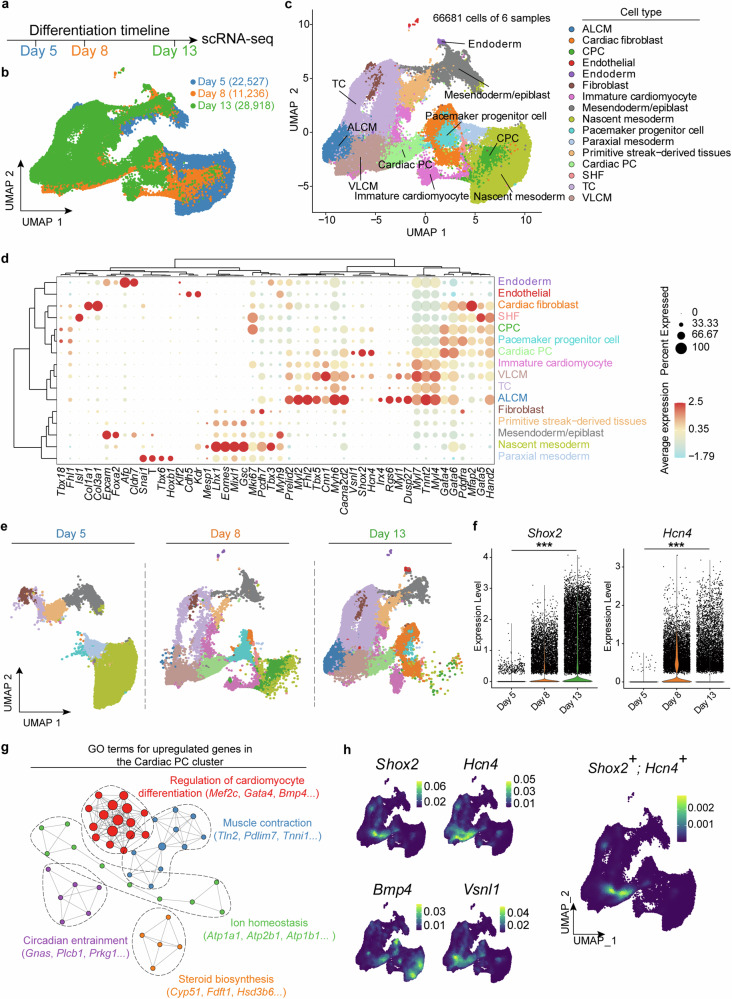
**Cardiac PC cluster was identified by scRNA-seq during in vitro cardiac PCs differentiation.** **a** Schematic representation of the timeline of sample collection for scRNA-seq (days 5, 8, and 13) (*n* = 2). **b** UMAP dimensionality reduction plot of samples on days 5, 8, and 13. 22,527, 11,236, and 28,918 cells were sequenced from days 5, 8, and 13 samples, respectively. **c** UMAP and clustering of scRNA-seq data identified 16 distinct clusters (VLCM, TC, SHF, Cardiac PC, Primitive streak-derived tissues, Paraxial mesoderm, Pacemaker progenitor cell, Nascent mesoderm, Mesendoderm/epiblast, Immature cardiomyocyte, Fibroblast, Endothelial, Endoderm, CPC, Cardiac fibroblast, and ALCM). Each dot represented an individual cell colored by an annotated cluster. TC transitional cell; SHF second heart field. **d** Dot plot of cell-specific markers defining each cluster. **e** UMAP dimensionality reduction plots divided by samples on days 5, 8, and 13. Different colors represent scRNA-seq clusters defined in (**c**, **d**). **f** Violin plots of the normalized unique molecular identifier (UMI) counts for two cardiac PC markers (*Shox2* and *Hcn4*) in days 5, 8, and 13 samples. Each dot in the plot represents one cell. The expression level means the log normalized data. Kruskal–Wallis rank sum test: *P* < 0.001 (***). **g** GO (gene ontology) term analysis for the genes upregulated in the Cardiac PC cluster. GO terms with *P* < 0.05 was considered significant. **h** UMAPs depicting the expression density of typical PC markers genes (*Hcn4*, *Shox2*, *Bmp4*, and *Vsnl1*) upregulated in the Cardiac PC cluster

To unravel the dynamic process of cardiac PCs differentiation induced by the FSK method, we utilized a droplet-mediated scRNA-seq platform (10× Genomics Chromium) to capture cells at different time points (days 5, 8, and 13), corresponding to the completion of mesoderm, cardiac PCs induction, and later stage maintenance respectively (Fig. 4a). We sequenced 81,599 cells at an average of about 50,000 reads per cell (Supplementary Table 1). After quality control, cells with aberrant gene detections (< 500 or > 6000 genes) were removed. Especially, we filtered cells with high mitochondrial gene coverage (> 10%) in day 5 samples because quality control of mitochondrial RNA (mtRNA) might introduce a bias that particularly discriminates cardiac PCs (days 8 and 13).^42^ In this manner, we sequenced 66,681 cells at three time points, with 22,527, 11,236, and 28,918 in day 5, 8, and 13 samples, respectively (Fig. 4b). Unsupervised clustering of the scRNA-seq data identified 23 distinct clusters (cluster 0–22, Supplementary Fig. S6a, b), which were annotated into 16 major cell types based on cell-specific markers (Fig. 4c, d): including Nascent mesoderm (Cluster 0, 1, and 12), Mesendoderm/epiblast (Cluster 9 and 17), Cardiac progenitor cell (CPC) (Cluster 16), Pacemaker progenitor cell (Cluster 10), cardiac PC (Cluster 8) as well as VLCM (Cluster 2 and 5), ALCM (Cluster 4) and non-cardiac cells.

As expected, the majority of cells on day 5 were mesodermal cells (Fig. 4e and Supplementary Fig. S6c). The clusters of Pacemaker progenitor cells and Cardiac PCs already emerged on day 8, consistent with our experimental observations (Fig. 2g). The number of mesoderm and pacemaker progenitor cells was significantly reduced on day 13 (Fig. 4e and Supplementary Fig. S6c), reflecting the sufficient differentiation from mesoderm to cardiac PC lineage. We next analyzed the expression of two cardiac PCs markers (*Shox2* and *Hcn4*). Although both *Shox2* and *Hcn4* were detected on day 8, the expression of both markers was higher on day 13 (Fig. 4f), consistent with the flow cytometry data, indicating that more cardiac PCs were generated and some cardiac PCs might be more mature on day 13. Besides, genes upregulated in the Cardiac PC cluster were enriched in the function of cardiomyocyte differentiation regulation, muscle contraction, ion homeostasis, and circadian entrainment, etc. (Fig. 4g). The Cardiac PC cluster not only highly expressed *Shox2* and *Hcn4* but also expressed abundant SAN and cardiac PC markers mentioned in previous research, such as *Vsnl1*, *Bmp4*, *Cacna2d2*, *Igfbp5*, *Lbh*, and *Dact1* (Fig. 4h and Supplementary Fig. S6d).^32,43^ Notably, the *Shox2*^+^*; Hcn4*^+^ cells were mainly in the Cardiac PC cluster, while the *Hcn4*^*+*^ cells were not restricted to this cluster (Fig. 4h), suggesting that *Hcn4* single positive might import a false-positive definition of cardiac PCs. To compare our differentiated cardiac PCs with the in vivo SAN cells, we integrated our data with the scRNA-seq data of E13.5 mouse SAN cells (GSE130461, Supplementary Fig. S6e).^44^ As indicated by the expression of *Shox2* and *Hcn4* markers, SAN cells in mice were highly overlapped with our differentiated cardiac PCs in Uniform Manifold Approximation and Projection (UMAP), illustrating that our in vitro differentiated cardiac PCs obtained from the FSK method were similar to in vivo PCs. Together, these results showed that dual positive markers (*Shox2* and *Hcn4*) could define cardiac PCs more precisely, and our in vitro cardiac PCs differentiation protocol, the FSK method, guided a credible pacemaker lineage.

### Cardiac PCs differentiation trajectory was successfully charted

**Fig. 5:**
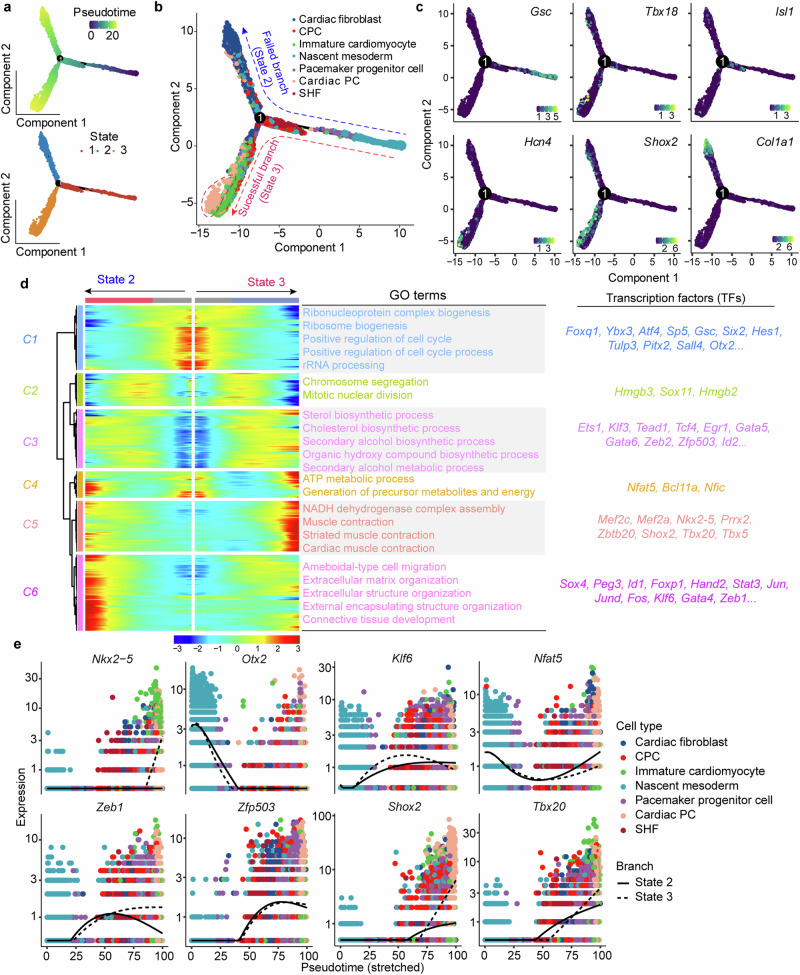
**Cardiac PCs differentiation trajectory was successfully charted.** **a** Unsupervised transcriptional trajectory of cardiac PCs differentiation, colored by pseudo-time and cell states (States 1–3). **b** Trajectory reconstruction of cardiac PCs differentiation revealed three branches: pre-branch (State 1), failed branch (State 2), and successful branch (State 3). Colors represented different cell clusters. **c** The expression of marker genes of three branches. Mesoderm markers: *Gsc*; cardiac PC markers: *Hcn4, Shox2, Tbx18*, and *Isl1*; cardiac fibroblast marker: *Col1a1*. **d** The differentially expressed genes along the pseudo-time were divided into six clusters (C1–C6) showing different patterns. The top GO terms of each cluster were shown. Highly expressed TFs of each gene expression cluster were shown. **e** The expression of representative genes (*Nkx2-5*, *Otx2*, *Klf6*, *Nfat5*, *Zeb1*, *Zfp503*, *Shox2*, and *Tbx20*) in the in the pseudo-time trajectory. The expression level means the log normalized data

To explore the refinements in cardiac PCs differentiation over time, we employed pseudo-time analysis to construct the differentiation trajectory with our scRNA-seq data (Fig. 5a). The trajectory depicted three different states, bifurcating from the main pre-branch (State 1) into two branches representing a successful branch to cardiac PCs (State 3) and a branch to cardiac fibroblasts (State 2) (Fig. 5a, b). *Gsc*, a mesoderm marker, was highly expressed in the initial stage of State 1 (Fig. 5c). The expression of *Isl1* and *Tbx18* at the crossroad of cardiac PCs differentiation trajectory suggested that the progenitor cells with posterior heart field (PHF) characters might contribute to cardiac PCs generation.^45^ Two cardiac PC markers (*Shox2* and *Hcn4*) indicated the successful branch to cardiac PCs in State 3, while the high expression of *Col1a1* represented another branch to cardiac fibroblasts (Fig. 5c).

To comprehensively view the differentiation trajectory, we analyzed 2829 differentially expressed genes (DEGs, with *q*-value < 0.01) and observed six gene expression clusters with different patterns (Fig. 5d). Genes in expression cluster 1 (C1) (a gene cluster referred to the mesoderm stage), enriched in ribosome biogenesis, were gradually downregulated from pre-branch (State 1), showing the translation activity was declining during the differentiation process (Fig. 5d). Conversely, the proliferation and sterol biosynthesis processes were upregulated (C2 and C3), especially in the middle stage of the differentiation trajectory (Fig. 5d), which might be due to the existence of progenitor cells, such as CPCs and pacemaker progenitor cells. Genes that increased along the State 3 trajectory were involved in the functions of NADH dehydrogenase complex assembly and muscle contraction (Fig. 5d), consistent with the nature of cardiac PCs.^46,47^ Notably, the failed branch (State 2) was highly enriched in extracellular matrix (ECM) organization (Fig. 5d, e), suggesting that the trajectory to cardiac fibroblasts might be a branch during our cardiac PCs differentiation. Since TFs are critical in the specification and differentiation of cardiac PCs,^48^ we further analyzed the expression of TFs in six gene expression clusters (Fig. 5d). TFs expressed in the mesoderm, such as *Gsc*, *Pitx2*, and *Hes1*, were highly expressed in C1. Several progenitor-related TFs (*Sox11*, *Ets1*, *Gata5*, and *Gata6*) were enriched in C2 and C3. *Tcf4*, *Zeb2*, *Zfp503*, and *Id2* were also highly expressed in C3, indicating their potential roles in cardiac PCs differentiation. C5 represented the state of cardiac PCs and consisted of abundant TFs expressed in PCs, such as *Mef2c*, *Shox2*, and *Tbx5*. Moreover, *Id1*, *Jun*, *Fos*, *Klf6*, *Gata4*, and *Zeb1* in C6 were closely related to the process of ECM, reflecting its trajectory to the cardiac fibroblasts. Figure 5e shows the representative TFs during the cardiac PCs differentiation. In summary, these data depicted the trajectory of cardiac PCs differentiation and revealed potential critical TFs for PCs development.

### ZFP503 functioned effectively in the cardiac PCs differentiation

**Fig. 6:**
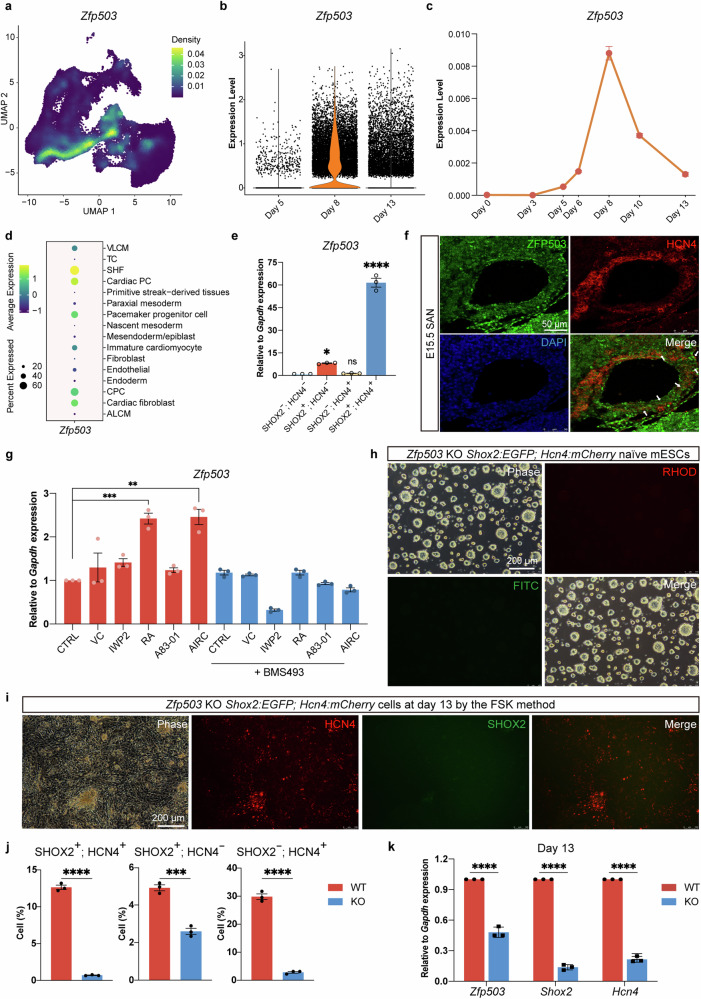
**ZFP503 functioned effectively in SHOX2**^**+**^**; HCN4**^**+**^ **cardiac PCs differentiation.** **a** UMAPs depicting the expression density of *Zfp503* in the scRNA-seq data. **b** Violin plot showed *Zfp503* expression level at different time points (days 5, 8, and 13) during cardiac PCs differentiation. The expression level means the log normalized data. **c** The dynamic change of *Zfp503* expression at different differentiation time points during cardiac PCs differentiation following the FSK method. Experiments were performed on *Shox2:EGFP; Hcn4:mCherry* mESC line. Relative to *Gapdh* expression. Data were presented as means ± SEM from technical triplicates (*n* = 3). **d** Dot plot of *Zfp503* in different cell clusters in scRNA-seq data. The size and color of the circles represent the gene expression percentage and average expression level, respectively. **e** qRT-PCR analysis revealed that day 13 SHOX2^+^; HCN4^+^ cells enriched *Zfp503* expression. Relative to *Gapdh* expression, normalized to SHOX2^−^; HCN4^−^ cells. Data were presented as means ± SEM from technical triplicates (*n* = 3). **f** Expression of ZFP503 in mouse SAN at E15.5. Arrows point to ZFP503^+^; HCN4^+^ cells. Scale bar: 50 μm. **g** The transcriptional expression of *Zfp503* on day 8 differentiation after 48 h treatment with different chemicals (0.5 mM Vc, 5 μM IWP2, 0.25 μM RA, 5 μM A83-01, 2 μM BMS493, or AIRC induction). CTRL, cells cultured in RB-i medium only during differentiation days 6–8. Relative to *Gapdh* expression, normalized to CTRL. Data were presented as means ± SEM from technical triplicates (*n* = 3). **h** Phase and live cell fluorescence images of *Zfp503* KO *Shox2:EGFP; Hcn4:mCherry* naïve mESC line (2i/LIF). Scale bar: 200 μm. **i** Live cell fluorescence images displayed impaired cardiac PCs differentiation in *Zfp503* KO *Shox2:EGFP; Hcn4:mCherry* mESCs by the FSK method on day 13. Scale bar: 200 μm. **j** Flow cytometry analysis showed *Zfp503* KO *Shox2:EGFP; Hcn4:mCherry* mESCs reduced cardiac PCs differentiation by the FSK method on day 13. WT represented *Zfp503*^+/+^ *Shox2:EGFP; Hcn4:mCherry* cells; KO represented *Zfp503* KO *Shox2:EGFP; Hcn4:mCherry* cells. Data were presented as means ± SEM from biological triplicates (*n* = 3). **k** qRT-PCR analysis of the expression of *Zfp503, Shox2*, and *Hcn4* on day 13 differentiation. Relative to *Gapdh* expression, normalized to differentiated cells on day 13 with *Zfp503*^*+/+*^ *Shox2:EGFP; Hcn4:mCherry* mESCs. WT represented *Zfp503*^+/+^ *Shox2:EGFP; Hcn4:mCherry* cells; KO represented *Zfp503* KO *Shox2:EGFP; Hcn4:mCherry* cells. Data were presented as means ± SEM from technical triplicates (*n* = 3). Comparisons between multiple groups were performed with a one-way ANOVA test (**e**, **j**, **k**). *P* values were calculated using a two-tailed student *t*-test to compare two groups of data (**g**). Statistical significance was indicated as follows: ns not significant; *P* < 0.05 (*); *P* < 0.01 (**); *P* < 0.001 (***); *P* < 0.0001 (****)

While reviewing the hit gene list from the single-cell transcriptome, a zinc finger TF, *Zfp503*, came to our attention. *Zfp503* barely expressed on day 5 while significantly increased on days 8 and 13 (Fig. 6a, b). The dynamic change of *Zfp503* expression in cardiac PCs differentiation was consistent with the pseudo-time analysis (Figs. 5e and 6c), which displayed that *Zfp503* expressed in the early period of the cardiac PCs developmental process, from SHF, CPCs, pacemaker progenitor cells to cardiac PCs (Fig. 6d). As an RA-related gene, *Zfp503* significantly enriched in the Cardiac PC cluster (Fig. 6d). Also, from scRNA-seq of E13.5 mouse SANs, *Zfp503* was enriched in primary PCs, implying its potential role in SAN development both in vivo and in vitro. High level of *Zfp503* in sorted SHOX2^+^; HCN4^+^ cells was validated by qRT-PCR and immunofluorescence staining (Fig. 6e and Supplementary Fig. S7a). Concomitantly, ZFP503 co-expressed with HCN4 in developing SAN of E15.5 mouse embryo (Fig. 6f and Supplementary Fig. S7b).

Previous findings defined *Zfp503* as a marker of neural progenitor cells and an RA-activated gene.^25,49^ The putative RA response element (RARE) is located 1600 bp downstream of the *Zfp503* transcriptional start site (TSS).^25^ Luciferase reporter assays further confirmed that RA activated the transcriptional activity of *Zfp503* (Supplementary Fig. S7c). While the RA signaling pathway is important in cardiac PCs’ fate determination, the relationship and mechanism of *Zfp503* and cardiac PCs differentiation need to be further elucidated. Not surprisingly, a low dose of RA (0.25 μM) activated *Zfp503* expression, whereas AIRC treatment (containing 0.25 μM RA) promoted a similar degree or slight increase of *Zfp503* expression (Fig. 6g). *Zfp503* was activated by RA in a dosage-dependent manner, which was repressed by BMS493 (Fig. 6g and Supplementary Fig. S7d). These data confirmed *Zfp503* as an RA target gene in the cardiac PCs differentiation.

We speculated that *Zfp503* respond to the RA signaling pathway and functions in the process of cardiac PCs development. *Zfp503* increased with RA treatment and functioned in the cardiac PCs developmental process and promoted the generation of SHOX2^+^; HCN4^+^ cells. RA boosted the effect of A83-01, IWP2, and Vc (called AIC) treatment and upregulated the efficiency of SHOX2^+^ ; HCN4^+^ cell differentiation (Supplementary Fig. S7e).

To further verify our speculation, a loss of function assay was performed by sgRNA targeting *Zfp503* exon 2 (Supplementary Fig. S7f). HM *Zfp503* KO *Shox2:EGFP; Hcn4:mCherry* mESC clones were identified by Sanger sequencing (Supplementary Fig. S7g). *Zfp503* KO mESCs displayed normal dome-shape mESC morphologies (Fig. 6h and Supplementary Fig. S7h). Then, we differentiated *Zfp503* KO mESCs into cardiac PCs by the FSK method. Strikingly, from *Zfp503* KO mESCs, fewer SHOX2^+^; HCN4^+^ cardiac PCs were produced, corroborated by low *Shox2* and *Hcn4* expression (Fig. 6i–k). These data suggested that *Zfp503* played an important role in cardiac PCs generationm and the loss of which was deleterious.

## Discussion

High-quality seeds are paramount for qualitative production. So far, most mouse PSCs differentiation methods start with cells cultured in serum/LIF, which are heterogeneous with cells biased to differentiation lineages.^50,51^ While various approaches have been attempted to optimize the signaling pathways to direct the differentiation routes, the quality of ‘seeds’, i.e., PSCs, was largely overlooked due to the limited culture media for PSCs. Recently, it has been proposed and verified along the embryo development, upon the exit from naïve pluripotency, the epiblast transiently goes through a FS to acquire differentiation potential before committing to a certain lineage. It has been demonstrated that FSCs could be induced to differentiate efficiently.^22,52^ Based on this progress in stem cell research, we established a new mouse cardiac PCs differentiation method by transitioning naïve PSCs to formative pluripotency for subsequent directed cardiac PCs differentiation, which produced SHOX2^+^; HCN4^+^ cells with typical PC characteristics, showcasing the applicability of our strategy.

Investigation on SAN development and in vitro cardiac PCs differentiation from PSCs have documented signaling pathways such as BMP, WNT, Activin/Nodal/TGF-β and RA, the crosstalk of which has essential roles in the differentiation route from mesoderm to cardiac PCs.^31,53–56^ Manipulating WNT signaling has been widely used in cardiac lineage differentiation from PSCs. Due to the difference in the application of WNT activation and suppression during various cardiac PCs differentiation methods, the critical role of WNT signaling in cardiac PCs differentiation is still not clearly defined.^31,34,38,57^ Yechikov et al. showed that inhibition of Activin/Nodal/TGF-β signaling pathway together with WNT pathway improved the efficiency of cardiac PCs differentiation.^58^ RA signaling pathway was also demonstrated to be critical in promoting atrial and SAN-like fate while inhibiting ventricular-like differentiation.^31,55,59^ Although Protze et al. showed that RA increased the level of cardiac PC markers but did not affect cardiac PCs differentiation efficiency, the use of RA in conjunction with BMP during mesoderm induction promoted cardiac PCs differentiation. Many other studies have also demonstrated that RA promoted the differentiation of PCs when combined with other signaling modulators.^55,59^ Therefore, we decided to include RA together with A83-01 and IWP2 in our protocol.

Interestingly, in our observation, the effects of RA, A83-01, and IWP2 alone were not significant in obtaining SHOX2^+^; HCN4^+^ cells. However, when combined, the number of SHOX2^+^; HCN4^+^ cells increased significantly, indicating the complex and delicate regulation of signaling pathways in cardiac PCs differentiation. On the other hand, the crosstalk among these pathways may direct the differentiation route of cardiac PCs^59^, whereas manipulating a single pathway was not significant. We, therefore, optimized the time and strength of either activating or repressing these pathways during cardiac PCs differentiation with small molecule combinations, which further increased the dual positive cells to more than 20%. By applying more specific and potent chemicals targeting the relevant pathway/component at the right differentiation window, more efficient cardiac PCs differentiation may be realized.

Because of the pluripotent character of PSCs, it is inevitable that the terminal differentiation culture is a mixture of various cell types. The purification of target cells has ultimate importance for characterization and cell therapy. Sorting by flow cytometry or magnetic beads based on fluorescence reporters or specific cell surface markers has been widely used for cell purification.^31,38,60,61^ Morikawa et al. successfully constructed an HCN4p-EGFP trans-gene mESC line for the first time and obtained about 0.9% EGFP^+^ cells with EB differentiation.^15,29^ In the present study, we achieved about 22.10% SHOX2^+^; HCN4^+^ and 32.43% SHOX2^−^; HCN4^+^ cells with the FSK method (Fig. 2g), and the dual positive cells increased 12 and 42 folds compared with EB (1.84%) and M10 (0.52%) methods, respectively. More importantly, electrophysiology and scRNA-seq showed that SHOX2^+^; HCN4^+^ cells were more similar to primary mouse PCs than the single positive cells.

The conservation of gene networks in SAN of different species including mouse and human has been characterized.^32,33^ However, physiologically, the heart rates for mice and humans are about 670 and 70 b.p.m., which is significantly different. The spontaneous rate is 4.6-times faster, and the action potential (AP) is 2.4-times shorter, while the *I*_*f*_ current is about 5-fold higher in mouse than in human, which may be caused by higher expression of ion channels such as HCN1, 2, 4, Cav3.1, RyR2 and SERCA2 in mouse SAN.^62^ Several laboratories have done pioneering work on human cardiac PCs (hPCs) differentiation that also laid a solid foundation for our current research. By modulating BMP, Activin/Nodal/TGF-β, and RA signaling pathways, Protze et al. induced human ESC (hESC) (HES3-NKX2-5^gfp/w^) into SAN-like pacemaker cells (SANLPCs), characterized by positive for pan-cardiomyocyte surface marker SIRPA and mesenchyme marker CD90, while negative for NKX2-5:GFP.^31^ More recently, Han et al. established *SHOX2:GFP; MYH6:mCherry* hESC line to produce hPCs.^37,61^ Using nine substances targeting singling pathways and epigenetic modifiers, about 46.6% SHOX2:GFP^+^ cells were produced, though only *SHOX2* is the cardiac PC-specific expression gene in this reporter system.^37^ Wakimizu et al. generated *HCN4-EGFP* transgenic, *SHOX2-mCherry* knock-in reporter human iPSC line, with different hPCs differentiation protocol, the dual positive cells ranged from 34%–44%. The gene editing strategies applied in this study may result in an N-terminal fusion protein or disruption of the coding sequence of *HCN4* and *SHOX2*.^63^ In the future, more cardiac PC-specific expression genes or gene combinations are needed for PCs purification and characterization.

sAP of cardiac PCs is the key to the development of cardiac BP technology. In this study, AP recording revealed that 55.00% SHOX2^+^; HCN4^+^ cells had typical cardiac PC-like APs and characteristic *I*_*f*_ current, which could increase by cAMP (Supplementary Fig. S5e, f) and inhibit by ivabradine (Fig. 3e and Supplementary Fig. S5i). Interestingly, in SHOX2^+^; HCN4^−^ and SHOX2^−^; HCN4^+^ subpopulations, 44.44% and 35.29% cells displayed cardiac PC-like APs, respectively (Fig. 3d). It is worth noting that there was still a 22.73% population of cardiac PCs in the SHOX2^−^; HCN4^−^ population. This might be attributed to our stringent cell sorting gates to obtain a relatively homogenous cell population. After cell sorting, low expression of *mCherry, Hcn4*, and *Vsnl1* was detected in SHOX2^−^; HCN4^−^ cells, which may express low levels of protein to enable some cells to display cardiac PC-like APs. These data suggested that using one cardiac PC marker alone is not reliable in PCs identification; on the other hand, by tracing the cell fate transition among these single and dual positive populations, we may further improve the cardiac PCs differentiation efficiency. In addition, we demonstrated that by MEA, cardiac PC aggregates acted as a dominant pacemaker to pace primary NMVMs. More critically, after cardiac implantation of cardiac PCs, they were capable of ectopically pacing the ventricle of rat heart ex vivo.

scRNA-seq facilitates us in understanding developmental trajectories and key factors determining cardiac PCs differentiation. The day 5 dataset revealed that most cells were mesendoderm progenitors, verifying the efficient commitment of FSCs after activation of WNT and Activin/Nodal/TGF-β signaling pathway.^22^ In subsequent small molecule cocktail-directed cardiac PCs differentiation, the trajectory showed that a portion of cardiac PCs was developed from *Isl1* and *Tbx18* expressing cells, indicating the in vitro development process of cardiac PCs may originate from progenitor cells with PHF properties, mirroring the development of bona fide cardiac PCs in vivo (Fig. 5c).^64,65^

Importantly, we validated *Zfp503*, a downstream target of RA signaling pathway,^49^ functioning in the development of cardiac PCs. It has been documented that *Zfp503* is involved in regulating early neuromesodermal progenitors and later brain and limb development.^25,66,67^ *Zfp503* is a potential player mediating the crosstalk of BMP, WNT, and RA signaling pathways.^67^ In our scRNA-seq dataset, *Zfp503* was expressed from SHF progenitor cells to cardiac PCs, which highly matched the developmental process of cardiac PCs. Moreover, *Zfp503* null mutation resulted in low efficiency of SHOX2^+^; HCN4^+^ cardiac PCs differentiation. RA appropriately induced *Zfp503* expression and promoted the differentiation of SHOX2^+^; HCN4^+^ cardiac PCs, consistent with previous reports that the RA signaling pathway promoted the development of cardiac PCs.^39,59^

The top DEGs of each cluster also indicated the co-existence of different cardiac progenitors. These results, on the one hand, suggested the possibility of efficiently obtaining atrial and ventricular cardiomyocytes with a modified FSK method. On the other hand, it will further direct us to improve the cardiac PCs differentiation efficiency. For example, *Nkx2-5* was highly expressed in the first heart field (FHF) and SHF clusters. It has been demonstrated the antagonism between *Nkx2-5* and *Shox2* in cardiac progenitors determined their commitment either to cardiomyocytes or to cardiac PCs.^9^ Therefore, by manipulating the cell fate transition, the yield of cardiac PCs may increase further.

In summary, we have established a simple and efficient platform for the generation of mouse cardiac PCs from PSCs. With scRNA-seq, we mapped the in vitro development trajectory of cardiac PCs and verified *Zfp503* as a key factor in guiding cardiac PCs differentiation. These findings will not only provide a high-quality cell source for cardiac BP but enhance our understanding of cardiac PCs development as well. By establishing specific reporter PSC lines, following the developing trajectory, and modulating the relevant signal pathways, our integrated cardiac PCs differentiation strategy may be extended to other cell types that are urgently needed in regenerative medicine.

## Materials and methods

### Ethics statement

All the animal experiments were approved by the Animal Care and Use Committee at the School of Medicine, Tongji University (No. TJBB00921701). Neonatal mice were euthanized by decapitation before harvesting hearts for cardiomyocyte isolation. For in vivo pilot transplantation, rats were intubated for mechanical ventilation followed by anesthesia with 4% isoflurane. For the isolation of adult hearts, rats were euthanized by cervical dislocation after anesthesia with 4% isoflurane. All procedures were performed in accordance with the Guide for the Care and Use of Laboratory Animals made by the U.S. National Institutes of Health.

### Animals

Neonatal C57/BL6J mice (1–3 day-old) and Sprague Dawley rats (male, 150–200 g, 6-week-old) were used in this study. All animals were purchased from Shanghai Sippe-Bk Lab Animal Co., Ltd., China.

### Cell culture

mESCs were cultured in a 0.2% gelatin-coated plate with 2i/LIF (leukemia inhibitory factor) medium and serum/LIF medium. mESCs were routinely passaged by Accutase or 0.25% trypsin–EDTA dissociation. Mycoplasma test was performed every 2 weeks and all cells used in this study were mycoplasma free.

### Generation of *Shox2:EGFP; Hcn4:mCherry* mESC reporter line

WT E14TG2a mESCs were transfected with spCas9, donor vector, and pGL3-U6-sgRNA-PGK-puromycin by Lipofectamine 3000. *mCherry* was first introduced into the *Hcn4* locus. After another 4 to 6 days, individual colonies were picked, expanded, and characterized by PCR amplification and subsequent sequencing for correct targeting. The same process was performed to introduce *2A-EGFP* into the *Shox2* locus in *Hcn4:mCherry* mESCs.

### Embryoid body (EB) method

mESCs cultured in serum/LIF were plated as hanging drops on the lid of a tissue culture dish with M10 medium (KnockOut DMEM plus 10% fetal bovine serum) supplemented with 0.5 mM Vc. Every drop contains 1000 cells in a volume of 20–30 µL. At day 5, EBs were collected and plated into a gelatin-coated 6-well plate. 5 µM IWR1-endo and 0.25 mM Vc were added into the M10 medium for 2 days to induce cardiac differentiation. The medium was switched to maintain medium (MEM supplemented with Insulin-Transferrin-Selenium-Sodium Pyruvate, penicillin streptomycin glutamine, and β-mercaptoethanol) for short-term cell culture (7–14 days) before analysis.

### M10 method

In total, 2 × 10^4^ mESCs cultured in serum/LIF were plated into one well of gelatin-coated 6-well plate overnight. The next day, the medium was switched to M10 containing 0.5 mM Vc. Two days later, CHIR (3 μM) was added to activate the WNT signaling pathway for 24 h. RPMI1640 supplemented with B27 minus insulin and 0.5 mM Vc (RBC-i) was used to differentiate the cells for 2 days. Then, cells were treated with 5 μM WNT inhibitor IWP2 for 48 h to promote cardiac lineage differentiation. The cells were maintained in RPMI1640 with B27 and Vc (RBC) till further analysis.

### FSK method

In total, 5 × 10^5^ naïve mESCs were plated into one well of fibronectin (10 µg/mL)-coated 6-well plate. The next day, the medium was switched to FSK medium containing N2B27 supplemented with 2 µM BMS493, 2 µM XAV939, 3–6 ng/mL Activin A, and 1% KSR. After 72 h, the cells were gently dissociated and replated at 5 × 10^5^ cells/well into fibronectin-coated 6-well plate, induced with mesoderm derivation medium, including N2B27 containing 20 ng/mL Activin A, 0.5 mM Vc and 3 µM CHIR (ACC) for 48 h. Subsequently, the medium was changed to RBC-i. After 24 h, cells were treated with 5 µM A83-01, 5 µM IWP2, 0.5 mM Vc, and 0.25 µM RA (AIRC) for 2 days with everyday medium change. After that, the medium was changed back to RBC-i for 48 h with everyday medium change. In order to promote cell proliferation, cells were cultured in RBC from day 10 onward, and then harvested on days 13 or 30 for subsequent analysis.

### Statistical analysis

All data represented three biological or technical replicates or more unless otherwise indicated in the figure legends. Statistical analyses were performed by GraphPad Prism Version 8.2.1(279) or R software (version 4.1.1). All data were presented as mean ± SEM. Error bars were indicated in the figure legends. All statistical analyses were conducted using a two-tailed student *t*-test, Mann-Whitney test, one-way ANOVA test, or Kruskal-Wallis rank sum test, where appropriate. *P* value < 0.05 was considered statistically significant. Statistical significance was indicated as follows: ns not significant; *P* < 0.05 (*); *P* < 0.01 (**); *P* < 0.001 (***); *P* < 0.0001 (****).

## Supplementary information

Supplementary Materials

Cell morphologies and physiological activities of day 13 SHOX2^+^; HCN4^+^ cardiac PCs

## Data Availability

The following public scRNA-seq dataset was used in this study: the scRNA-seq dataset (GSE130461) of E13.5 *Shox2*^*Cre/+*^*; R26R*^*mTmG*^ SAN cells including atrial cells.^44^ The raw data of scRNA-seq presented in this study have been deposited in the Genome Sequence Archive (GSA) in National Genomics Data Center under the accession number CRA010142. The codes used for scRNA-seq analysis can be found at: https://github.com/bowenlin0512/Cardiac_PCs_project.
