# Supplementary material for: A new paradigm for generating high-quality cardiac pacemaker cells from mouse pluripotent stem cells
Source: Signal Transduct Target Ther. 2024 Sep 6;9:230. doi: 10.1038/s41392-024-01942-w (PMC11377569; doi:10.1038/s41392-024-01942-w)
Supplement: Supplementary file 1 — Supplementary Materials [file 41392_2024_1942_MOESM1_ESM.docx]

Supplementary Materials for

**A new paradigm for generating high-quality cardiac pacemaker cells from mouse pluripotent stem cells**

Zheyi Lin^1,2,3,4,5,6^, Bowen Lin^1,2,3,4,5,6^, Chengwen Hang^1,2,3,4,5,6^, Renhong Lu^1,2,3,4,5,6^, Hui Xiong^1,2,3,4,6,7^, Junyang Liu^1,2,3,4,6,7^, Siyu Wang^1,2^, Zheng Gong^1,2^, Mingshuai Zhang^1,2,3,4,6,7^, Desheng Li^1,2,3,4,6^, Guojian Fang^1,2,3,4,5,6^, Jie Ding^1,2,3,4,5,6^, Xuling Su^1,2^, Huixin Guo^8^, Dan Shi^1,2,3,4,6^, Duanyang Xie^1,2,3,4,5,6^, Yi Liu^1,2,3,4,5,6^, Dandan Liang^1,2,3,4,5,6,9^, Jian Yang^1,2,3,4,6,7,9✉^ & Yi-Han Chen^1,2,3,4,5,6,9✉^

Correspondence to: jy279@tongji.edu.cn; yihanchen@tongji.edu.cn

**This PDF file includes:**

Materials and Methods

Supplementary References

Supplementary Figures. S1 to S7

Supplementary Tables. S1 to S2

Caption for Supplementary Movie. S1

**Other Supplementary Materials for this manuscript include the following:**

Movie. S1

**Materials and Methods**

*Cell culture*

mESCs were cultured in 0.2% gelatin-coated plates with 2i/LIF medium, i.e. N2B27 medium, supplemented with 3 µM CHIR99021 (CHIR, GSK3β inhibitor), 1 µM PD0325901 (MEK inhibitor) and 10 ng/mL LIF. To make 500 mL N2B27 medium, 250 mL DMEM/F12 medium (Gibco) and 250 mL Neurobasal medium (Gibco) were supplemented with 5 mL penicillin streptomycin glutamine (PSG) (Gibco), 5 mL N2 supplement (homemade), 10 mL B27 supplement (Gibco) and 0.1 mM β-mercaptoethanol. To culture mESCs in serum/LIF medium, 500 mL Knock-Out DMEM (Gibco) was supplemented with 15% fetal bovine serum (FBS), 10 ng/mL LIF, 5 mL PSG and 0.1 mM β-mercaptoethanol. For mESCs differentiation in M10 serum medium (500 mL KnockOut DMEM supplemented with 10% FBS, 5 mL PSG and 0.1 mM β-mercaptoethanol), LIF was removed from the medium.

*Generation of Shox2:EGFP; Hcn4:mCherry mESC reporter line*

*Shox2* single guide RNA (sgRNA) sequence for knock-in: 5’-GTACAAGTAATGA CGCCGGC-3’. *Hcn4* sgRNA sequence for knock-in: 5’-GAGCTGTACAAGTAAT GAGC-3’. The sgRNA was cloned into pGL3-U6-PGK-puromycin vector to generate constructs. 800 bp-long fragments upstream and downstream of the stop codon of selected genes were PCR amplified from genomic DNA as 5’ and 3’ homology arms respectively. The homology arms and fluorescence genes (*2A-EGFP* and *mCherry*) were cloned into pMD20-T vector (Takara) to generate donor vector.

In detail, 5 X 10^5^ wild-type E14TG2a mESCs were transfected with 2 µg spCas9, 2 µg donor vector and 0.7 µg pGL3-U6-sgRNA-PGK-puromycin by Lipofectamine 3000. After transfection, the cells were selected with puromycin (1 µg/mL) for 4 days. CRISPR/Cas9 off-target site prediction was performed on the CCTop and the Cas-OFFinder websites.^1,2^

*Flow cytometry analysis and cell sorting*

For flow cytometry analysis, single cell suspension was prepared by treating the cells with collagenase II (0.5 µg/mL) for 30 min at 37℃, then washed and resuspended in PBS. Cells were loaded onto Beckman Flow cytometer CytoFLEX. Data analysis was performed with FlowJo software. For samples requiring antibody immunostaining, the protocol is described below.

For cell sorting, the preparation of single cell suspension is the same as flow cytometry analysis. Resuspension buffer is RPMI1640 with 1% FBS. Cells were sorted on a Beckman Coulter MoFlo Astrios 4 and gathered through four independent channels. Cells were sorted into RBC medium containing 10% FBS. The sorted cells were harvested for RNA extraction or cultured in RBC plus with 10%-20% KSR for further analysis.

*Immunofluorescence*

Cells were fixed with 4% paraformaldehyde (PFA) for 15 min, and washed 3 times with PBS at room temperature. After fixation, samples were blocked by PBS with 0.1% Triton X-100 (PBST), 3% BSA, and 1% goal serum for 20 min, incubated with primary antibodies overnight at 4℃. The next day, cells were washed with PBST and incubated with fluorescence conjugated secondary antibody for 1 h in dark at room temperature. Then, after washing with PBST, cell nuclei were stained with DAPI and observed under fluorescence microscope.

For tissue staining, after optical mapping, the apex of heart was dissected, then fixed in 4% PFA overnight. Hereafter, sections (8 µm) were cut from paraffin-embedded apex for further immunofluorescence staining. Briefly, after de-paraffinization, rehydration and antigen retrieval. Sections were blocked in PBST with 10% normal goat serum for 20 min, and followed by primary antibody incubation at 4℃ overnight. The next day, slides were washed in PBST and incubated for 1 h in fluorochrome-conjugated secondary antibody in the dark. Finally, autofluorescence quencher and fluoroshield with DAPI were used to reduce autofluorescence, visualize the nuclei and mount sections. The sections were observed under fluorescence microscope.

*Whole-cell patch clamp recordings*

Spontaneous action potential (sAP) of individual sorted cells was recorded by whole-cell patch clamp. 2-4 X 10^4^ sorted cells were plated into growth factor reduced Matrigel-coated 35 mm dishes filled with 2 mL cardiac PCs plating medium (contains 45% conditioned RBC medium, 45% fresh RBC medium and 10% KSR). 24-48 h later, cells were washed with PBS once and cultured with fresh RBC media plus 5%-10% KSR. Medium was changed every 3 days. In the follow 2-12 days, APs of cells were recorded by patch clamp recordings. The whole process was oxygenated and performed around 35℃. According to standard patch-clamp techniques, APs were recorded in the current clamp mode by borosilicate glass microelectrode filled with the pipette solution. Tip resistance was kept at a range of 8-12 MΩ.

For *I_f_* current recording, Tyrode’s solution contains 1 mM Ba^2+^ to block inward rectifier potassium current (*I_K1_*). A voltage-clamp mode was applied. Holding potential of -35 mV and eliciting *I_f_* by a family of voltage steps from -10 mV to -120 mV for 1.5 s with 10 mV increment. *I_f_* density was measured as ivabradine-sensitive current (1 μM). For cAMP-actived *I_f_* current recording, 1 mM cAMP was added into the intracellular solution before experiment.

Standard Tyrode’s solution contains the following components: 135 mM NaCl, 10 mM glucose, 5.4 mM KCl, 1.8 mM CaCl_2_, 0.3 mM Na_2_HPO_4_, 0.3 mM KH_2_PO_4_, 10 mM HEPES (pH 7.4, adjusted with NaOH). The standard pipette solution consists of: 140 mM KCl, 10 mM EGTA, 10 mM HEPES, 5 mM glucose, 3 mM Na_2_ATP (pH 7.2, adjusted with KOH). All the data were collected through an EPC-10 amplifier (HEKA, Germany), Axon MultiClamp 700B (Molecular Devices, USA) and Axon Digidata 1550B (Molecular Devices, USA) and analyzed with Patchmaster 2.42 (HEKA, Germany) and Clampfit 10.7 (Molecular Devices, USA).

*In vivo pilot transplantation and ex vivo optical mapping under a Langendorff heart preparation*

A total of 15 Sprague Dawley rats (male, 150-200 g, 6-week-old) were used as the recipients. Ten rats were injected with differentiated cells (days 13-17). Others were injected with the corresponding buffer. Rats were intubated for mechanical ventilation followed by anesthesia with 4% isoflurane. Cells were dissociated into small clumps with EDTA for 10 min, then resuspended in RBC medium with Matrigel on ice. Thoracotomy of the left parasternal region exposed the apex of the left ventricle. Approximately 1-2 X 10^6^ cells were injected into the left ventricular apex of rat with an insulin syringe (29G needle). To prevent immune rejection, animals were treated with Cyclosporine A (15 mg/kg) and Methylprednisolone (2 mg/kg) by intraperitoneal injection.

To assess *in vivo* pacemaker capacity of the injected cells, *ex vivo* optical mapping was performed 4-6 days post-transplantation. The optical mapping under a Langendorff heart preparation was carried out as previously described.^3^ Hearts were rapidly harvested from anesthetized (4% isoflurane) and heparinized (10 U/g) rats after cervical dislocation. Subsequently, the heart was retrogradely perfused with oxygenized standard Tyrode’s solution through the aorta at a constant temperature (35 ± 2℃). The heart resumed contractile function in seconds, followed by the voltage-sensitive dye RH237 (1-2 μM) staining for 5 min and excess dye was washed away for another 5 min. During perfusion, blebbistatin (10 µM) were added into Tyrode’s solution in order to uncouple excitation-contraction and then eliminate motion artifacts. MiCam ULTIMA imaging system (SciMedia) attached to the stereoscopy imaging system (Leica) was used to detect the change of membrane potential. The filter excited the stained heart at a wavelength of 505 ± 20 nm. High-resolution optical fluorescence images were collected at a speed of 500 frames/s in the left apex of heart. After acquiring adequate baseline, Adenosine (6 mg/mL, 0.1 mL), Methacholine (1 μM, 0.1 mL) and Lidocaine (0.005%, 0.1 mL) were used to create reversible short-term atrioventricular (AV) block 1 to 2 times. The optical images were captured to examine the normal conduction pattern of the left ventricle and the ectopic rhythm from transplanted site. Data analysis was performed with BV_Ana software (SciMedia).

*Isolation of* *neonatal mouse ventricular myocytes (NMVMs)*

Primary NMVMs were isolated from neonatal C57/BL6J mice following previous protocol with modifications.^4^ Briefly, neonatal mice (1-3 day-old) were decapitated, then the hearts were dissected, washed with cold PBS and cut into pieces. Tissues were dissociated in PBS with 0.25% trypsin at 4℃ for 6 h, followed by PBS with 0.1% collagenase type II and 1% BSA at 37℃ for 10 min. Repeat the collagenase II digestion procedure 2 to 3 times. The supernatant was collected and supplemented with 10% FBS, then filtered with 100 μm cell strainer to remove incompletely digested cell clumps. Filtered cell suspension was centrifuged at 1,000 rpm for 10  min, resuspended in DMEM with 10% FBS, plated in uncoated dishes for 1.5 h at 37℃ to remove fibroblasts. Cell supernatant was collected for further experiments.

*Multiple microelectrode array (MEA) assay*

Approximately 3-5 X 10^4^ sorted cells were resuspended in RBC + 10% KSR, plated into 96-well Clear Round Bottom Ultra-Low Attachment Microplate to form aggregates by low-speed centrifugation at 100 g, and cultured for 3 days.

1-1.2 X 10^5^ NMVMs and one aggregate were plated into one well of Matrigel-coated 24-well CytoView MEA Plate. After 3 days, cells would grow confluent and form a monolayer, then MEA assay was performed from days 5-8. The culture environment was under the control of the MEA and impedance system (Axion BioSystems, Maestro Pro multi-well MEA platform). Data were analyzed by Axls Navigator 3.2 software (Axion BioSystems).

*RNA extraction and quantitative reverse transcriptase PCR (qRT-PCR)*

Total RNA of cultured cells was extracted using guanidinium thiocyanate-phenol-chloroform extraction and Direct-zol RNA Microprep Kit. For sorted cells, total RNA extraction was isolated and purified by GenElute Single Cell RNA Purification Kit. First-strand cDNA was synthesized with HiScript III RT SuperMix for qRT-PCR kit. qRT-PCR was performed on the Applied Biosystems SteponePlus Real-Time PCR System 6 (Thermo Fisher) with SYBR green method according to the manufacturer’s instructions. Gene expression was normalized to *Gapdh*. All primers used for qRT-PCR are listed in **Supplementary Table S2**.

*Vector construction and luciferase* *reporter assay*

The sequence 5’-TGACCCTCCAATCACCTTGACCCTCCAATCACCTTGACCCT CCAATCACCT-3’ from *Zfp503* locus containing RA response element (RARE) was synthesized (*Zfp503*-RARE). 3 X *Zfp503*-RARE was assembled into pGL3-basic vector containing an SV40 promoter to generate luciferase reporter vector (pGL3-*Zfp503*p-Luc). E14TG2a ESCs were plated at a density of 5 X 10^4^ cells/well in 12-well plates. The next day, 1 μg pGL3-*Zfp503*p-Luc and 0.1 μg *Renilla* per well were transfected into mESCs with Lipofectamine 3000. 24 h after transfection, the medium was replaced with N2B27 minus RA (with and without 1 μM RA) for 2 days. Then the cells were harvested to measure luciferase activity with the Dual-glo Luciferase Assay System (Promega, E1910).

*Single cell RNA sequencing (scRNA-seq) and analysis*

Cell detachment was performed by Accutase, collagenase II (0.5 mg/mL) or trypsin-EDTA (0.125 %). All scRNA-seq libraries were prepared using Chromium Next GEM Single Cell 3’ Reagent Kits (v3.1) according to the manufacturer’s instruction. In brief, the nuclei were isolated into droplets together with gel beads coated with unique primers bearing 10 X cell barcodes, unique molecular identifiers (UMIs), and poly(dT) sequences. Barcoded cDNAs were generated by reverse transcription after disruption of emulsions and cDNAs were cleaned with DynaBeads MyOne Silane Beads (Thermo Fisher). After cDNA amplification and cleanup, 3’ gene expression libraries were constructed using reagents from Chromium Next GEM Single Cell 3’ Reagent Kits (v3.1). The final libraries were sequenced on the Illumina Novaseq 6000 platform with at least 45,000 mean reads per cell.

Sample demultiplexing, barcode processing and single-cell gene counting were performed by the Cell Ranger Single-Cell Software Suit (v4.0.0). Sequencing reads were aligned to the mouse reference genome (mm10) using STAR (v2.7.8a). Downstream analyses were performed by the Seurat R package (v4.0.5). Since quality control of mitochondrial RNA (mtRNA) might introduce a bias that particularly discriminates cardiac PCs,^5^ we only filtered the cells with higher than 4,000 or lower than 200 detected genes. Potential doublets were filtered using the DoubletFinder (v2.0.3) R package. After quality filtering, the expression matrics were normalized and scaled using ‘NormalizeData()’, ‘FindVariableFeatures()’, ‘ScaleData()’ and ‘RunPCA’ functions in Seurat R package step by step. All the samples were integrated by Harmony (v0.1.0) through ‘RunHarmony()’ function. To visualize our scRNA-seq data, we created a UMAP with ‘RunUMAP()’, for which we used the first 30 dimensions from the Harmony output with the reduction equals to ‘harmony’. Differentially expressed genes (DEGs) and maker genes of each cluster were identified by ‘FindAllMarkers’ function in the Seurat package with the parameters ‘min.pct = 0.25, logfc.threshold = 0.25’. Gene ontology (GO) analyses were conducted by clusterProfiler (v3.16.0) R package. Pseudo-time analysis was performed by Monocle (v2.20.0).

**Supplementary References:**

1 Bae, S., Park, J. & Kim, J. S. Cas-OFFinder: a fast and versatile algorithm that searches for potential off-target sites of Cas9 RNA-guided endonucleases. *Bioinformatics* **30**, 1473-1475 (2014).

2 Stemmer, M. et al. CCTop: An Intuitive, Flexible and Reliable CRISPR/Cas9 Target Prediction Tool. *PLoS One* **10**, e0124633 (2015).

3 Motayagheni, N. Modified Langendorff technique for mouse heart cannulation: Improved heart quality and decreased risk of ischemia. *MethodsX* **4**, 508-512 (2017).

4 Chen, Y. et al. Long Non-coding RNA ECRAR Triggers Post-natal Myocardial Regeneration by Activating ERK1/2 Signaling. *Mol Ther* **27**, 29-45 (2019).

5 Galow, A. M. et al. Quality control in scRNA-Seq can discriminate pacemaker cells: the mtRNA bias. *Cell Mol Life Sci* **78**, 6585-6592 (2021).

**Supplementary Figures:**

**
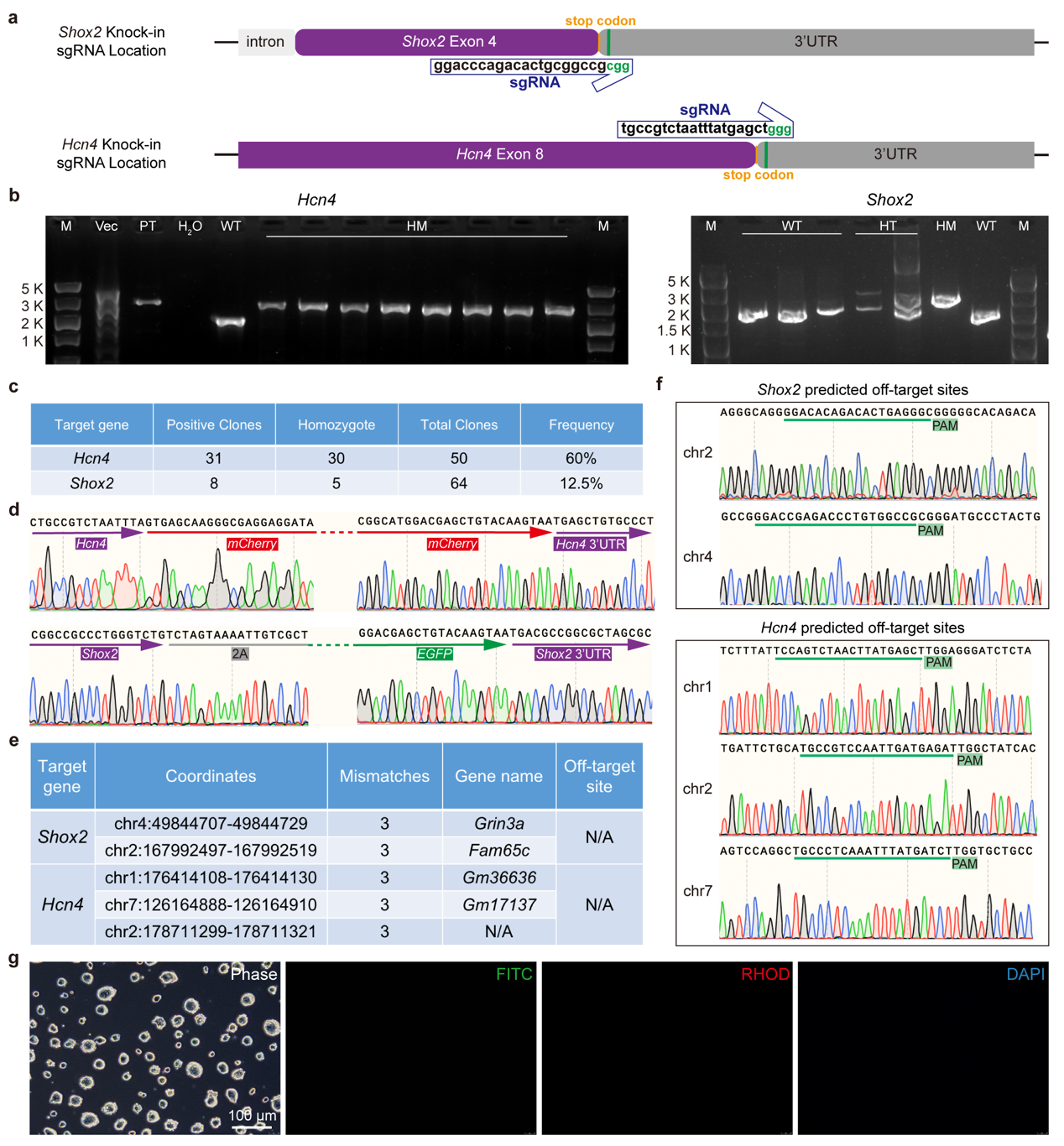
**

**Supplementary Figure. S1. Verification of *Shox2:EGFP; Hcn4:mCherry* mESC reporter line.**

1. Schematic illustration of sgRNAs location in *Shox2* and *Hcn4* loci for *Shox2:EGFP; Hcn4:mCherry* mESC reporter line construction.
2. Representative PCR genotyping results showed homozygotes, heterozygotes, and wild-type mESC clones picked. Left, agarose gel image of *Hcn4:mCherry* knock-in mESC line; right, agarose gel image of *Shox2:EGFP* knock-in mESC line construction. *Shox2:EGFP* knock-in mESC line construction was performed on the homozygous *Hcn4:mCherry* knock-in mESC clone (#55). Vec, *Hcn4:mCherry* knock-in donor vector; PT, positive control; WT, wild type; HM, homozygotes; HT, heterozygotes; M, 100-5000 bp DNA ladder.
3. The knock-in efficiency of *Hcn4:mCherry* and *Shox2:EGFP* by CRISPR/Cas9.
4. The sequencing results revealed that the sequence of *mCherry* and *2A-EGFP* inserted in-frame into the targeted loci of *Hcn4* and *Shox2,* respectively.
5. Summary of predicted gene editing off-target sites.
6. Representative sequencing results revealed no off-targets in *Shox2:EGFP; Hcn4:mCherry* mESC reporter line.
7. Live images of *Shox2:EGFP; Hcn4:mCherry* mESC reporter line cultured in 2i/LIF. The fluorescence channels (FITC, RHOD, and DAPI) showed no detectable fluorescence signals in *Shox2:EGFP; Hcn4:mCherry* mESC reporter line at the naïve state. Scale bar: 100 μm.

**
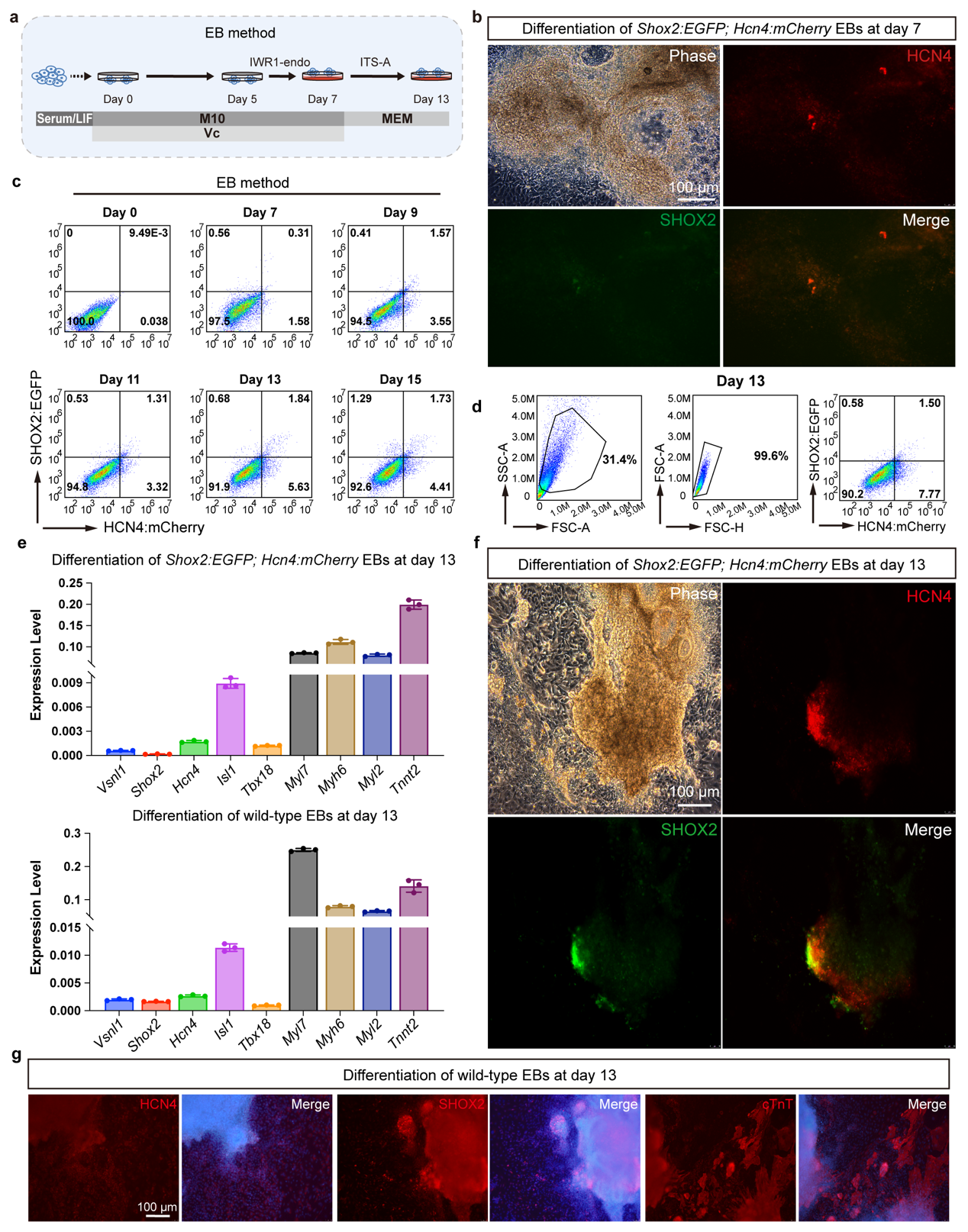
 Supplementary Figure. S2. EB method did not produce a sufficient number of cardiac PCs.**

1. Diagram of the EB method for cardiac PCs differentiation. mESCs cultured in serum/LIF medium were used as seed cells. ITS-A, Insulin-Transferrin-Selenium-Sodium Pyruvate.
2. Live cell images revealed that differentiation induced by the EB method generated a small amount of SHOX2^+^; HCN4^+^ cells on day 7 in *Shox2:EGFP; Hcn4:mCherry* mESC reporter line. Scale bar: 100 µm.
3. Representative flow cytometry analysis showed the distribution of fluorescent cells during cardiac PCs differentiation on days 0, 7, 9, 11, 13, and 15 by the EB method. Data were presented as one representative data from biological triplicates (*n* = 3).
4. Gating strategy of analyzing SHOX2^+^; HCN4^+^, SHOX2^+^; HCN4^−^, SHOX2^−^; HCN4^+^, SHOX2^−^; HCN4^−^ cells from day 13 differentiated cells by the EB method. The diagrams were presented as one representative experiment from biological repeats.
5. Transcriptional expression of different cardiac lineage markers. Experiments were performed on *Shox2;EGFP; Hcn4:mCherry* and wild-type EBs (day 13). Relative to *Gapdh* expression. Upper, *Shox2;EGFP; Hcn4:mCherry* EBs; lower, wild-type EBs. Cardiac PC markers: *Vsnl1, Shox2, Hcn4, Isl1*, and *Tbx18;* atrial markers: *Myl7* and *Myh6*; ventricular markers: *Myl2*; pan cardiac marker: *Tnnt2.* Data were presented as means ± SEM from technical triplicates (*n* = 3).
6. Representative live cell images showed the expression pattern of HCN4 and SHOX2 in *Shox2:EGFP; Hcn4:mCherry* EBs on day 13 differentiation. Scale bar: 100 µm.
7. Immunofluorescence images displayed the expression pattern of cardiomyocyte and cardiac PC markers in wild-type EBs (day 13). Cardiac PC markers: HCN4 and SHOX2; pan cardiac marker: cTnT. Scale bar: 100 μm.


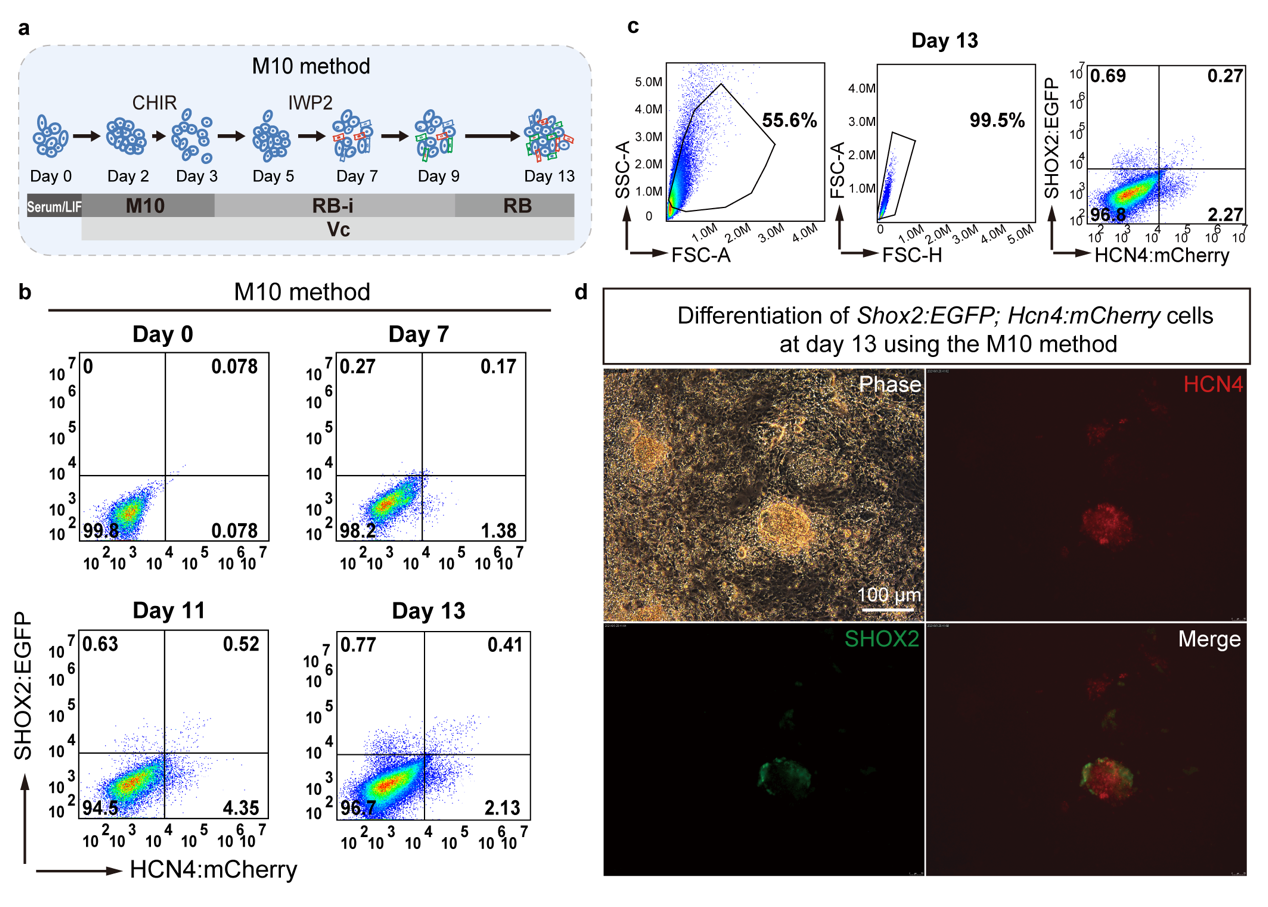


**Supplementary Figure. S3. M10 method did not produce a sufficient number of cardiac PCs.**

1. Diagram of the M10 method. mESCs cultured in serum/LIF medium were used as seed cells. RB-i, RPMI1640 plus B27 minus insulin; RB, RPMI1640 plus B27.
2. Representative flow cytometry analysis showed the distribution of fluorescent cells during cardiac PCs differentiation on days 0, 7, 11, and 13 by the M10 method. Data were presented as one representative data from biological triplicates (*n* = 3).
3. Gating strategy of analyzing SHOX2^+^; HCN4^+^, SHOX2^+^; HCN4^−^, SHOX2^−^; HCN4^+^, SHOX2^−^; HCN4^−^ cells from day 13 differentiated cells by the M10 method. The diagrams were presented as one representative experiment from biological repeats.
4. Representative live cell images displayed *Shox2:EGFP; Hcn4:mCherry* cells differentiated with the M10 method on day 13. Scale bar: 100 µm.

**
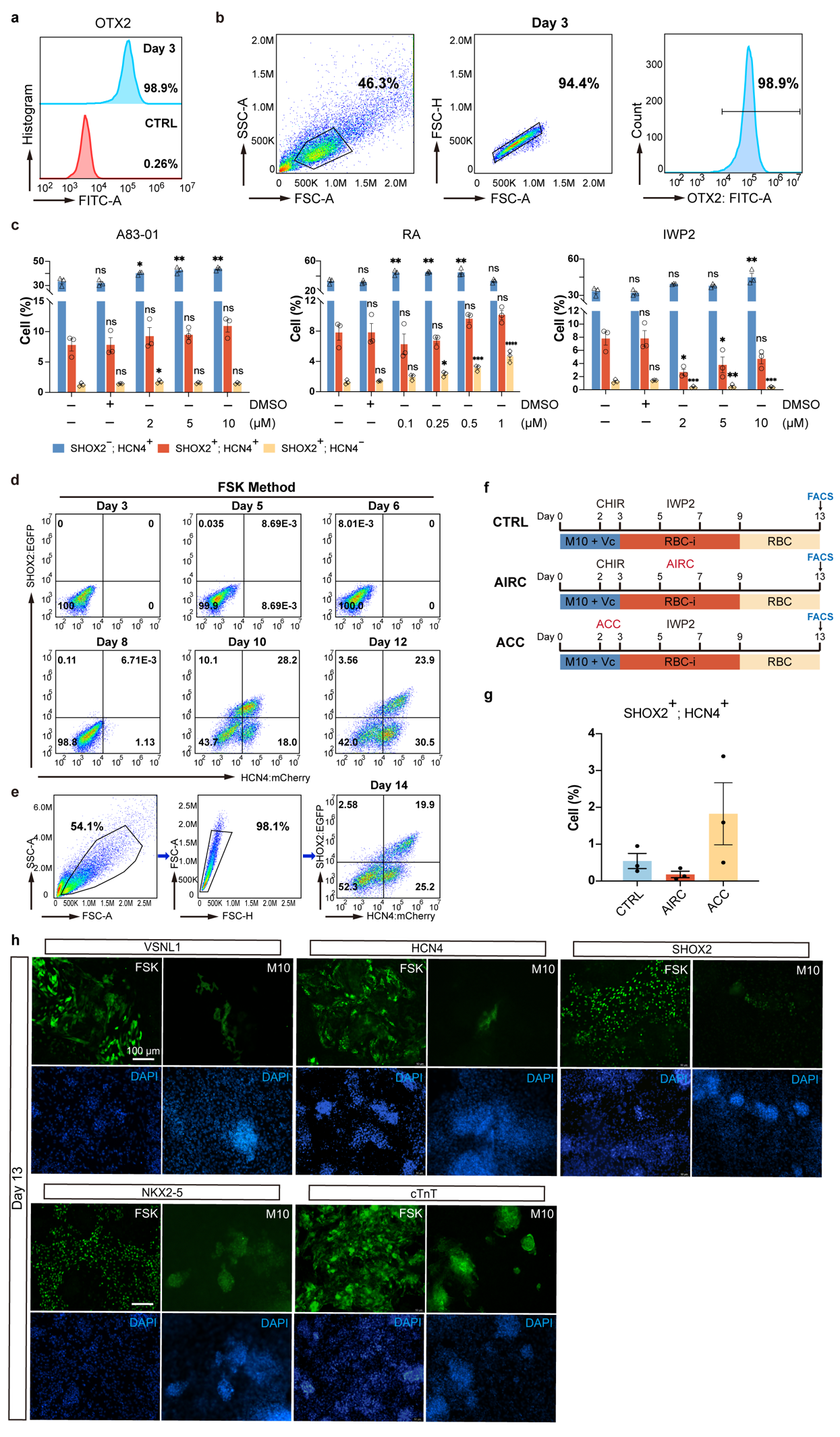
**

**Supplementary Figure. S4. Efficient generation of cardiac PCs with the FSK method.**

1. Representative flow cytometry analysis showed OTX2 highly expressed after formative state conversion on differentiation day 3. CTRL represented the unstained cells on differentiation day 3. Data were presented as one representative data from biological triplicates (*n* = 3).
2. Gating strategy of analysing OTX2^+^ cells from day 3 differentiated cells using the FSK method. Similar gating strategy was applied for analysing T^+^ and MESP1^+^ cells from days 4 to 6 differentiated cells using the FSK method.
3. Effects of different signaling pathways-related small molecules on cardiac PCs differentiation. Different doses of RA, A83-01, and IWP2 were added on from days 6-8, and flow cytometry analysis was performed on day 13. Blue, SHOX2^−^; HCN4^+^ cells; red, SHOX2^+^; HCN4^+^ cells; yellow, SHOX2^+^; HCN4^−^ cells. Data were presented as means ± SEM from biological triplicates (*n* = 3). *P* values were calculated with One-way ANOVA test. Statistical significance was indicated as follows: ns, not significant; *P* < 0.05 (*); *P* < 0.01 (**); *P* < 0.001 (***); *P* < 0.0001 (****).
4. Flow cytometry analysis showed the distribution of fluorescent cells during cardiac PCs differentiation on days 3, 5, 6, 8, 10, 12, and 14 by the FSK method. Data were presented as representative data from biological triplicates (*n* = 3).
5. Gating strategy of analyzing SHOX2^+^; HCN4^+^, SHOX2^+^; HCN4^−^, SHOX2^−^; HCN4^+^, SHOX2^−^; HCN4^−^ cells from day 14 differentiated cells by the FSK method, as shown in (*d*). The diagrams were presented as one representative experiment from biological repeats.
6. Optimization of the M10 method by ACC and AIRC induction. The scale of SHOX2^+^; HCN4^+^ subpopulation was determined by flow cytometry analysis on day 13. CTRL was the original M10 method. ACC and AIRC replaced CHIR and IWP2 treatment respectively. FACS, flow cytometry analysis.
7. Flow cytometry analysis of SHOX2^+^; HCN4^+^ subpopulation from day 13 differentiation cells. The experiment design was shown in (*f*). Data were presented as means ± SEM from biological triplicates (*n* = 3).
8. Immunofluorescence images displayed the expression pattern of cardiac PC and cardiac lineage markers in the FSK method using wild-type mESCs (day 13). Cardiac PC markers: VSNL1, HCN4, and SHOX2; cardiac lineage marker: NKX2-5 and cTnT. Scale bar: 100 µm.

**
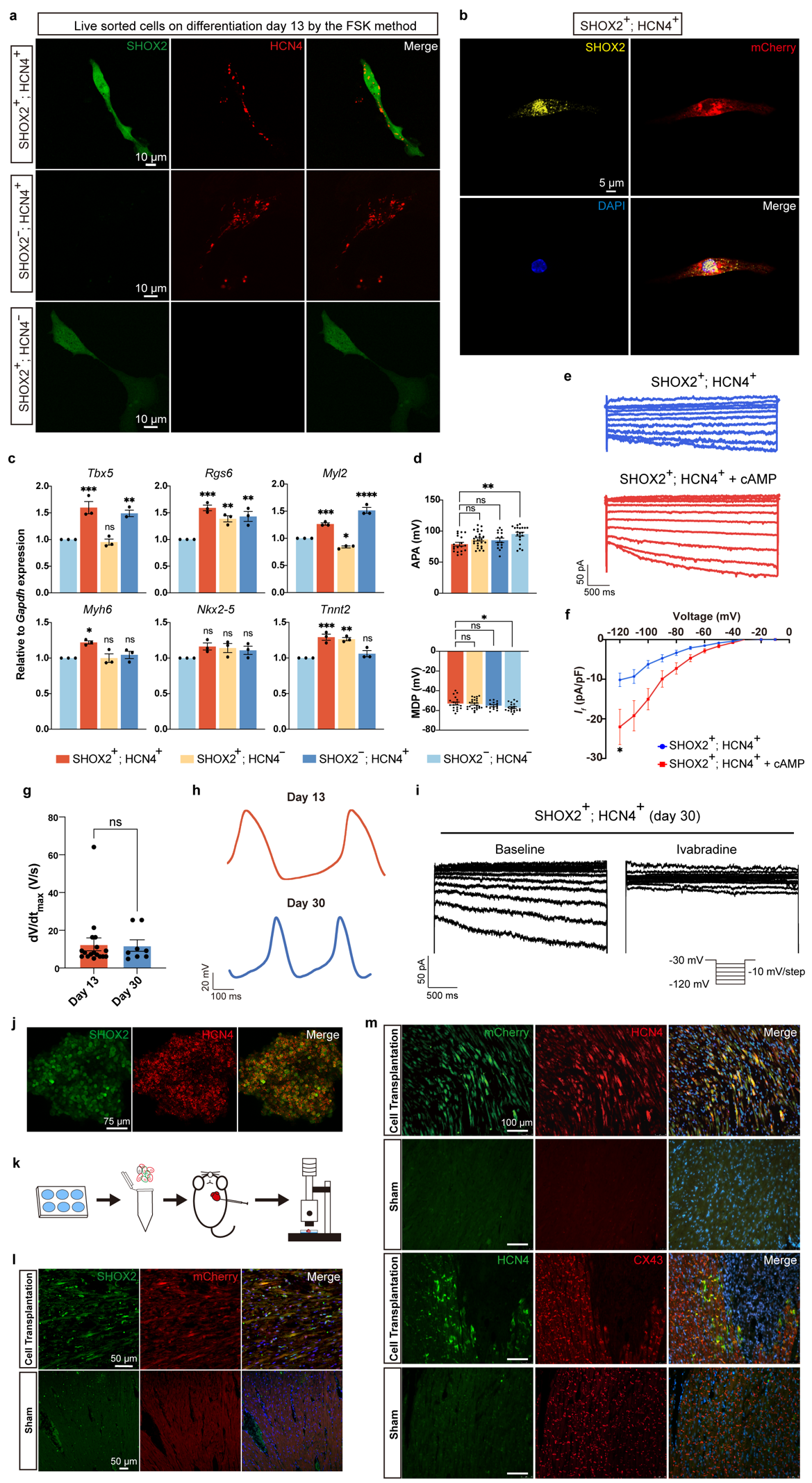
**

**Supplementary Figure. S5. SHOX2^+^; HCN4^+^ cells displayed cardiac PC** **characteristics.**

1. Confocal live cell images of three cell subpopulations sorted on differentiation day 13 using the FSK method. Upper, SHOX2^+^; HCN4^+^ cell; middle, SHOX2^−^; HCN4^+^ cell; lower, SHOX2^+^; HCN4^−^ cell. Scale bar: 10 µm.
2. Immunofluorescence staining showed the localization of SHOX2 and HCN4:mCherry in SHOX2^+^; HCN4^+^ cells sorted on differentiation day 13 using the FSK method. Scale bar: 5 µm.
3. qRT-PCR analysis of the expression of different cardiac subtype marker genes in different cell clusters. Relative to *Gapdh* expression, normalized to SHOX2^−^; HCN4^−^ cells. Cardiac PC markers: *Tbx5* and *Rgs6*; VLCM marker: *Myl2*; ALCM marker: *Myh6*; cardiac lineage markers: *Nkx2-5* and *Tnnt2*. Data were presented as means ± SEM from technical triplicates (*n* = 3).
4. Action potential amplitude (APA) and maximum diastolic potential (MDP) of four cell subpopulations on day 13 by the FSK method. Data were presented as means ± SEM from biological triplicates. Red, SHOX2^+^; HCN4^+^ cells (*n* = 20); yellow, SHOX2^+^; HCN4^−^ cells (*n* = 27); deep blue, SHOX2^−^; HCN4^+^ cells (*n* = 17); light blue, SHOX2^−^; HCN4^−^ cells (*n* = 22).
5. Representative *I_f_* current recorded from day 13 SHOX2^+^; HCN4^+^ cells treated without or with 1 mM cAMP.
6. Current-voltage relationship for *I_f_* current densities in day 13 SHOX2^+^; HCN4^+^ cells treated without or with 1 mM cAMP. Data were presented as means ± SEM from biological triplicates (*n* >= 5).
7. The maximum upstroke velocities recorded in day 13 and day 30 SHOX2^+^; HCN4^+^ cells. Data were presented as means ± SEM from biological triplicates (*n* >= 8).
8. Representative sAP recording of day 13 and day 30 SHOX2^+^; HCN4^+^ cells.
9. Representative recordings of *I_f_* current in day 30 SHOX2^+^; HCN4^+^ cells made at different membrane potentials in Tyrode’s solution with 1 mM Ba^2+^ and in the presence of the *I_f_* blocker ivabradine.
10. SHOX2 and HCN4 expression in cardiac PC aggregates formed by differentiation day 13 SHOX2^+^; HCN4^+^ cells. SHOX2 and HCN4 were maintained in cardiac PC aggregates. Scale bar: 75 μm.
11. Diagram of ectopic pacemaker implantation and optical mapping experiments.
12. Immunohistochemistry staining of SHOX2 and HCN4:mCherry in adjacent sections from the left ventricle of rat with transplanted cells. Scale bar: 50 μm.
13. Immunofluorescence images of paraffin section from the apex of rat heart with cell transplantation and sham surgery served as control. Scale bar: 100 μm.

Action potential recording and *I_f_* current recording were performed on Axon MultiClamp 700B and Axon Digidata 1550B (*e-i*). Comparisons between multiple groups were performed with One-way ANOVA test (*c*) and Kruskal-Wallis rank sum test (*d*). *P* values were calculated using Mann-Whitney test to compare two groups of data (*f*). Two-tailed Student’s *t*-test was performed (*g*). Statistical significance was indicated as follows: ns, not significant; *P* < 0.05 (*); *P* < 0.01 (**); *P* < 0.001 (***); *P* < 0.0001 (****).

**
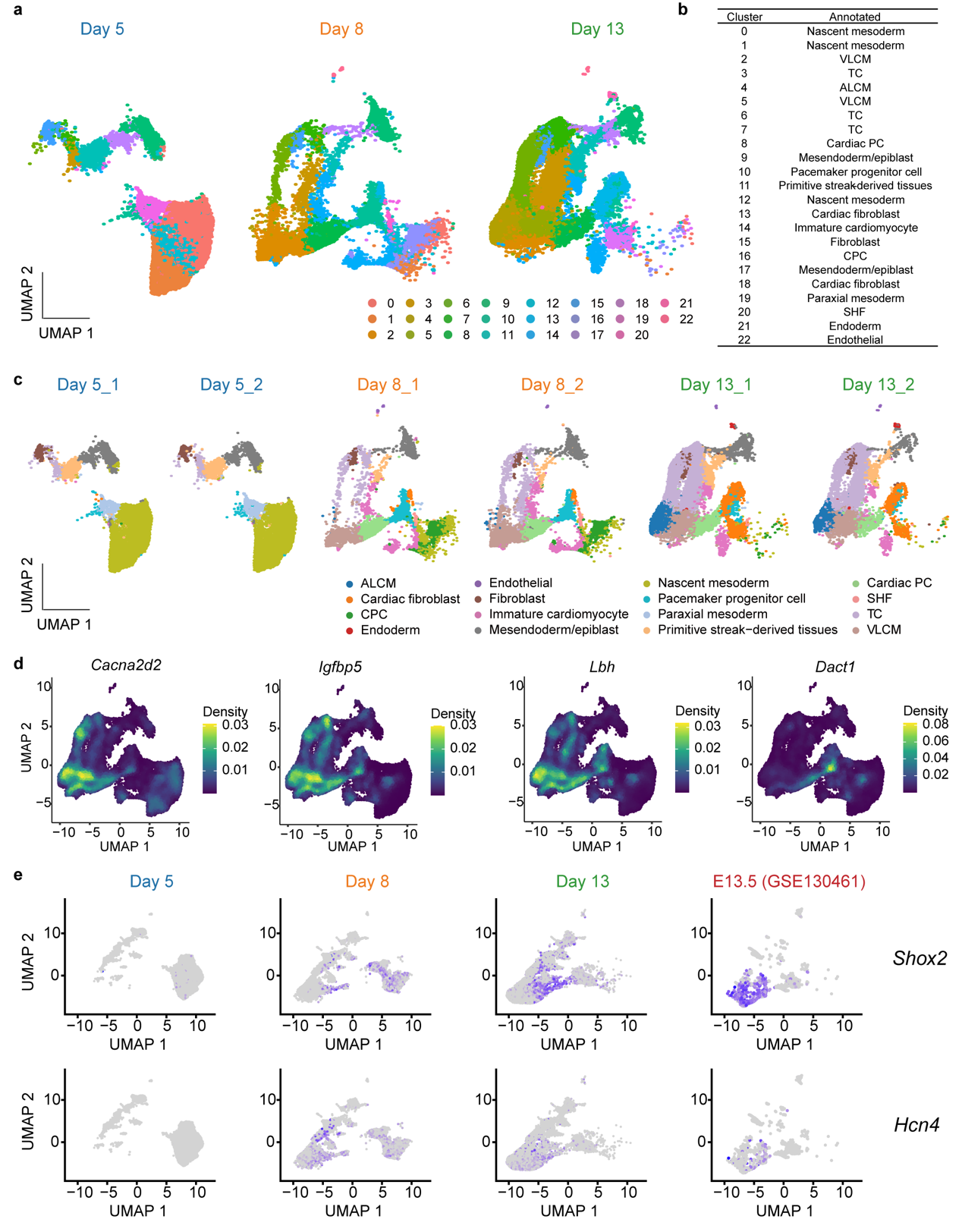
Supplementary Figure. S6. scRNA-seq analysis of *in vitro* cardiac PCs differentiation using the FSK method.**

1. UMAP and clustering of scRNA-seq data identified 23 distinct clusters.
2. 23 clusters in (*a*) were annotated according to different markers. Then, the sample cell clusters were merged in the subsequent analysis.
3. UMAP dimensionality reduction plots show different samples and biological duplications on days 5, 8, and 13.
4. The expression density of typical PC marker genes upregulated in the Cardiac PC cluster.
5. UMAP plots of *Hcn4* and *Shox2* expression on days 5, 8,13 and E13.5 mouse SAN (GSE130461). Our scRNA-seq data (days 5, 8, and 13 differentiated cells obtained from the FSK method in our study) were integrated with public E13.5 mouse SAN (GSE130461) scRNA-seq data. The purple dots were the positive expression cells.

**
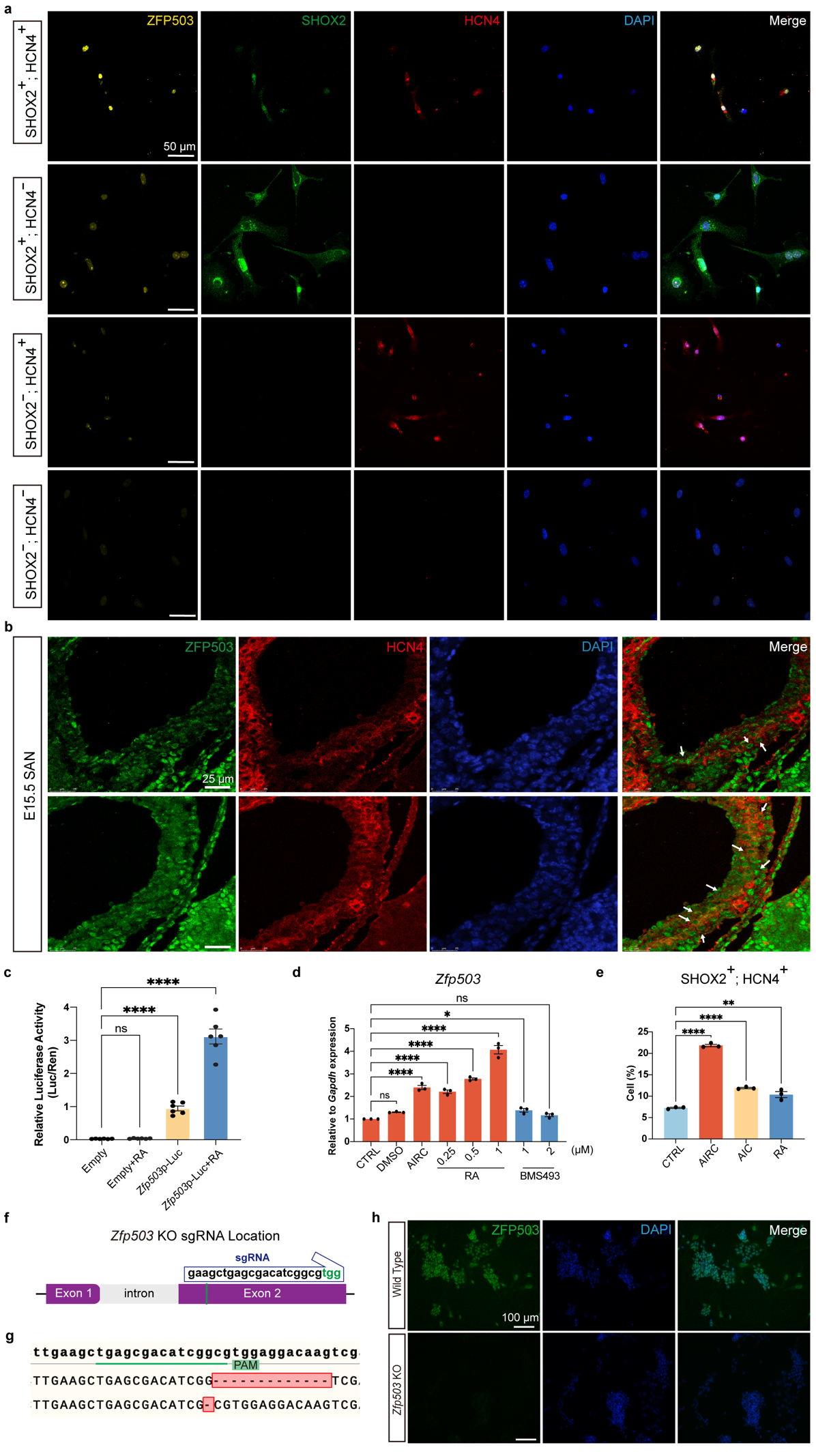
Supplementary Figure. S7. *Zfp503* functioned effectively in the differentiation process of cardiac PCs.**

1. Immunofluorescence staining showed the expression of ZFP503 in different cell types (SHOX2^+^; HCN4^+^, SHOX2^+^; HCN4^−^, SHOX2^−^; HCN4^+^, SHOX2^−^; HCN4^−^) sorted on differentiation day 13 by flow cytometry. Scale bar: 50 μm.
2. Expression of ZFP503 in mouse SAN at E15.5. Arrows point to ZFP503^+^; HCN4^+^ cells. Scale bar: 25 µm.
3. Luciferase reporter assays indicated *Zfp503* was an RA response gene. Wild-type E14TG2a mESCs were transfected with pGL3-*Zfp503*p-Luc or empty (pGL3-Basic-Luc) vector together with *Renilla* vector. The assays were performed after transfected E14TG2a cells were treated with or without 1 µM RA in N2B27 minus RA medium for 2 days. Data were presented as means ± SEM from biological triplicates (*n* = 6). Luc, firefly luciferase; Ren, *Renilla* luciferase.
4. qRT-PCR analysis revealed dosage-dependent activation of *Zfp503* expression on day 8 differentiation after 48 h treatment with different chemicals (0.5 mM Vc, 5 μM IWP2, 0.25μM RA, 5 μM A83-01, 2 μM BMS493, or AIRC induction). CTRL, cells cultured in RB-i medium only during differentiation days 6-8. Relative to *Gapdh* expression, normalized to CTRL. Data were presented as means ± SEM from technical triplicates (*n* = 3).
5. Flow cytometry analysis of SHOX2^+^; HCN4^+^ cells on day 13 by the FSK method. Cardiac PCs differentiation was induced by different chemical combination treatment from days 6 to 8. CTRL, RBC-i only on days 6-8; AIRC, combined treatment of A83-01, IWP2, RA, and Vc on days 6-8; AIC, combined treatment of A83-01, IWP2, and Vc on days 6-8; RA, RA alone on days 6-8. Data were presented as means ± SEM from biological triplicates (*n* = 3).
6. The location of sgRNA on exon 2 of *Zfp503* for gene knock-out (KO).
7. The Sanger sequence result of *Zfp503* KO *Shox2:EGFP; Hcn4:mCherry* mESC line. The PAM and sgRNA location were marked.
8. Immunofluorescence assay of wild type and *Zfp503* KO cells. RA (2 µM) was used to induce *Zfp503* expression by 48 h treatment. Scale bar: 100 μm.

Comparisons between multiple groups were performed with One-way ANOVA test (*c-e*). Statistical significance was indicated as follows: ns, not significant; *P* < 0.05 (*); *P* < 0.01 (**); *P* < 0.0001 (****).

**Supplementary Tables:**

**Table S1. The sequencing information of samples for scRNA-seq.**

| Sample | Estimated Number of Cells | Mean Reads per Cell | Median Genes per Cell |
| --- | --- | --- | --- |
| FSK_d5_1 | **13,589** | **48,592** | **4,055** |
| FSK_d5_2 | **13,511** | **45,627** | **4,030** |
| FSK_d8_1 | **9,210** | **68,029** | **4,579** |
| FSK_d8_2 | **10,192** | **56,434** | **4,073** |
| FSK_d13_1 | **24,455** | **46,695** | **3,892** |
| FSK_d13_2 | **10,642** | **50,789** | **3,208** |

**Table S2. List of qRT-PCR primer sequences.**

| Target gene | Sequence (5’-3’) |
| --- | --- |
| *Cacna2d2*-F | GCCGTATTTGCTGCCACTGATG |
| *Cacna2d2*-R | TATCCAGGCTGCGACGGTAGAA |
| *Cx40*-F | GTGCCAAACCAGGAGCAGATTC |
| *Cx40*-R | CGCCGTTTGTCACTATGGTAGC |
| *Cx43*-F | GGTGATGAACAGTCTGCCTTTCG |
| *Cx43*-R | GTGAGCCAAGTACAGGAGTGTG |
| *Esrrb*-F | GACATTGCCTCTGGCTACCACT |
| *Esrrb*-R | ACTTGCGCCTCCGTTTGGTGAT |
| *Fgf5*-F | GGCTCGGAACATAGCAGTTT |
| *Fgf5*-R | CGTGGGAGCCATTGACTTT |
| *Flk1*-F | GTGTGACCAAGAGTGACCAA |
| *Flk1*-R | ATCCCACTACCGAAAGCAATAA |
| *Foxa2*-F | AAGCCCGAGCACCATTAC |
| *Foxa2*-R | GTAGGCCTTGAGGTCCATTT |
| *Gapdh*-F | CGTGCCGCCTGGAGAAAC |
| *Gapdh*-R | AGTGGGAGTTGCTGTTGAAGTC |
| *Gata4*-F | GTGGCCTCTATCACAAGATGAA |
| *Gata4*-R | GCTCACCCTCGGCATTAC |
| *Gata5*-F | TCTGGCATCTCCAGTGTGTGCT |
| *Gata5*-R | ATGGACGAGGAGGTGAAGGCAA |
| *Gata6*-F | ATGCGGTCTCTACAGCAAGATGA |
| *Gata6*-R | CGCCATAAGGTAGTGGTTGTGG |
| *EGFP*-F | GAACCGCATCGAGCTGAA |
| *EGFP*-R | TGCTTGTCGGCCATGATATAG |
| *Hcn1*-F | TTCTCAGTCTCTTGCGGTTATT |
| *Hcn1*-R | CATCATGCCAATGAGGTTGAAG |
| *Hcn2*-F | CTACAGCGACTTCAGGTTCTAC |
| *Hcn2*-R | GATGCCCACGGGAATGATAA |
| *Hcn4*-F | CGTGCTCACTAAGGGCAACAAAG |
| *Hcn4*-R | GCACCTCATTGAAGTTGTCCACG |
| *Hhex*-F | CGGTCAAGTGAGGTTCTCCAAC |
| *Hhex*-R | CTCGGCGATTCTGAAACCAGGT |
| *Isl1*-F | TGAGGGTTTCTCCGGATTTG |
| *Isl1*-R | TCCCTAACAAAGCACGTACAG |
| *Klf2*-F | CACCTAAAGGCGCATCTGCGTA |
| *Klf2*-R | GTGACCTGTGTGCTTTCGGTAG |
| *Klf4*-F | CTATGCAGGCTGTGGCAAAACC |
| *Klf4*-R | TTGCGGTAGTGCCTGGTCAGTT |

| Target gene | Sequence (5’-3’) |
| --- | --- |
| *mCherry*-F | TGAGGTCAAGACCACCTACA |
| *mCherry*-R | CTGTTCCACGATGGTGTAGTC |
| *Mesp1*-F | TACGCAGAAACAGCATCCC |
| *Mesp1*-R | GCATGTCGCTGCTGAAGA |
| *Mly2*-F | AAGGTGTTTGATCCCGAGGG |
| *Mly2*-R | GGGAAAGGCTGCGAACATCT |
| *Mly7*-F | TGACCCAGGCAGACAAGTTC |
| *Mly7*-R | CGTGGGTGATGATGTAGCAG |
| *Myh6*-F | GCTGGAAGATGAGTGCTCAGAG |
| *Myh6*-R | CCAGCCATCTCCTCTGTTAGGT |
| *Nanog*-F | GCAAGAACTCTCCTCCAT |
| *Nanog*-R | ATACTCCACTGGTGCTGA |
| *Nkx2-5*-F | CCAACAGCAACTTCGTGAAC |
| *Nkx2-5*-R | TGGAGACGCCCGAATTG |
| *Nppa*-F | TACAGTGCGGTGTCCAACACAG |
| *Nppa*-R | TGCTTCCTCAGTCTGCTCACTC |
| *Oct4*-F | AAGCTGCTGAAGCAGAAGAG |
| *Oct4*-R | TTAAGGCTGAGCTGCAAGG |
| *Oct6*-F | AGTTCGCCAAGCAGTTCA |
| *Oct6*-R | CGAGAACACGTTACCGTAGAG |
| *Otx2*-F | GTCTGACCACTTCGGGTATG |
| *Otx2*-R | CGAGCTGTGCCCTAGTAAAT |
| *Prdm14*-F | CAGCGACTTCATTGCCAAAGGAG |
| *Prdm14*-R | GCCGTCGATAAAATGGCTCAGG |
| *Rex1*-F | GAGACTGAGGAAGATGGCTTCC |
| *Rex1*-R | CTGGCGAGAAAGGTTTTGCTCC |
| *Rgs6*-F | CAGTTCCTCAGATTCCTGGAGTC |
| *Rgs6*-R | CTCTTGCCAGATTTCCTCCACC |
| *Scn5a*-F | AGAGCGGCTGTGAAGATTT |
| *Scn5a*-R | CGGTGAAGGTGTACTCAACATA |
| *Shox2*-F | CTTTCATGCGCGAGGAATTG |
| *Shox2*-R | GGCTCCTATAAGGACACCTTTG |
| *Smoc2*-F | TGCAATGCCATCCATCCA |
| *Smoc2*-R | GGGCTGTGTTGTCACACTTA |
| *Sox17*-F | TTGGCCGCGTCCATAAA |
| *Sox17*-R | GACCTAACCCTTCGCCTAATC |
| *Sox2*-F | AACGGCAGCTACAGCATGATGC |
| *Sox2*-R | CGAGCTGGTCATGGAGTTGTAC |

| Target gene | Sequence (5’-3’) |
| --- | --- |
| *T*-F | TTCCCGAGACCCAGTTCATA |
| *T*-R | TGGCATCAAGGAAGGCTTTAG |
| *Tbx18*-F | CATTCTGGAGGCCATCACTAC |
| *Tbx18*-R | TTGGAGCAGAGCTGAAGAAC |
| *Tbx20*-F | ACTGGGCACGGAGATGATAA |
| *Tbx20*-R | GACTCAGGATCCACTCCAGAAA |
| *Tbx3*-F | GAAGTCAGGAAGGCGAATGT |
| *Tbx3*-R | ACAGTCGTCAGCAGCTATAATG |
| *Tbx5*-F | GCTTTTATCGCTGTGACTTCGTAC |
| *Tbx5*-R | GTAACTCCAGGTCATCACTGCC |
| *Tfcp2L1*-F | CAGCCTCTATCCAGGATGCACA |
| *Tfcp2L1*-R | CTCTGGACATCTTCAGGAGGTC |
| *Tnnt2*-F | GCTACAGACTCTGATCGAGGCT |
| *Tnnt2*-R | GCTCATTGCGAATACGCTGCTG |
| *Vsnl1*-F | GTGACGGCAAGATCACCCG |
| *Vsnl1*-R | AGGCCATCCTCATTCATTTTCAT |
| *Zfp503*-F | TGAAGATGCTGACGGCACGGAC |
| *Zfp503*-R | GGCTTCCCTATCTGCGAACATG |

**Supplementary Video:**

**Movie S1. Cell morphologies and physiological activities of day 13 SHOX2^+^; HCN4^+^ cardiac PCs.**
